# XLVI REUNIÓN ANUAL DE LA SOCIEDAD ANDALUZA DE NEUROLOGÍA

**DOI:** 10.31083/RN39503

**Published:** 2025-08-26

## Comunicaciones Orales

**CO-01.- Reducción Manifiesta de los Síntomas NO-motores en Pacientes con Enfermedad de Parkinson Tras Estimulación Bilateral Theta Burst Intermitente Sobre la Corteza Motora Primaria**

Paloma Macías García^1^, Raúl Rashid López^1^, F. Luis Sánchez Fernández^1^, Esteban Sarrias Arrabal^1^, Álvaro J. Cruz Gómez^1^, Javier J. González Rosa^1^

^1^H. U. Puerta del Mar, 11009 Cádiz, España

**Objetivos**: Investigar los mecanismos neurales subyacentes a la aplicación de estimulación magnética transcraneal theta burst intermitente (iTBS) sobre la corteza motora primaria (M1) como terapia complementaria para aliviar los síntomas no-motores de la enfermedad de Parkinson (EP).

**Material y Métodos**: 17 pacientes con EP participaron en un estudio de diseño cruzado, aleatorizado, doble-ciego y efecto placebo controlado. Se aplicó iTBS bilateralmente sobre M1 durante 5 días consecutivos (1 sesión/día). Los cambios en los síntomas no-motores a nivel cognitivo y neuropsiquiátrico fueron evaluados mediante exhaustiva batería neuropsiquiátrica, considerado el resultado principal de este estudio. Paralelamente, se recopilaron medidas de cambio en la excitabilidad corticoespinal mediante el registro de los umbrales motores en reposo y activo, así como en la actividad funcional en estado de reposo (fMRI).

**Resultados**: Hubo una mejoría en la sintomatología no-motora relacionada con el estado de ánimo (depresión y ansiedad) tras la aplicación de iTBS real. Este cambio fue igualmente observado tras recibir iTBS sham, aunque en menor grado. El porcentaje de pacientes respondedores para los síntomas del estado de ánimo (mejoría no-motora de acuerdo con el índice de Diferencia Mínima Clínicamente Importante) fue superior tras recibir iTBS real. Respecto a las medidas secundarias, tras recibir iTBS real, se observó un aumento de excitabilidad corticoespinal en el hemisferio más afectado, así como cambios sutiles en la actividad cerebral funcional en estado de reposo.

**Conclusiones**: La aplicación de 5 sesiones de iTBS bilateral sobre M1 pueden resultar efectivas para el alivio de la sintomatología ansioso-depresiva en la EP.

**CO-02.- Síndrome de Vasoconstricción Cerebral Reversible. Serie de 10 Casos**

Guevara Sánchez Eva^1^, Gallo Pineda Félix^1^, Milán Pinilla Rodrigo José^1^, Arjona Padillo Antonio^1^, Ruiz Franco María Luisa^1^

^1^H. U. Torrecárdenas, 04009 Almería, España

**Objetivos**: El Síndrome de Vasoconstricción Cerebral Reversible (SVCR) se caracteriza por la aparición de cefalea súbita y déficits neurológicos focales, asociados a la presencia de hemorragia o isquemia, y vasoconstricción segmentaria que se resuelve en un periodo de 3 meses. El objetivo de nuestro estudio es describir las características de una serie de pacientes con SVCR valorados en nuestro centro.

**Material y Métodos**: Búsqueda en el sistema Informático de nuestro hospital desde 2006 a 2022. Se incluyeron todos los pacientes con código CMBD para SVCR. Criterios de inclusión: mayor de 18 años, clínica compatible, vasoconstricción en neuroimagen, normalización completa o sustancial de la vasoconstricción en seguimiento y ausencia de diagnósticos alternativos.

**Resultados**: Obtuvimos una muestra final de 10 casos. 80% mujeres con edad media de 51.9. El factor de riesgo que más se relacionó con la presentación de esta entidad fue el tabaco presentándose en 4 pacientes (40%). La cefalea en trueno fue el síntoma común (100%) seguido del déficit neurológico (70%). El método diagnóstico principal fue la Ultrasonografía Doppler (100%) y la localización del vasoespasmo se dio mayoritariamente en la Arteria Cerebral Media (80%). La complicación más frecuente fue el ictus isquémico (60%).

**Conclusiones**: El SVCR es una entidad probablemente infradiagnosticada en nuestro hospital dada su prevalencia descrita previamente. En nuestra serie la ausencia de un desencadenante fue frecuente y la Ultrasonografía Doppler fue el método diagnóstico más utilizado, por lo que su realización, u otro método para demostrar la vasoconstricción, debería extenderse en pacientes con cefalea en trueno.

**CO-03.- Predictores Radiológicos y Séricos del Declive Cognitivo y la Discapacidad en Pacientes con Esclerosis Múltiple de Reciente Diagnóstico: Un Estudio Longitudinal de 3 Años**

Álvaro Javier Cruz Gómez^1^, Lucía Forero^1^, Fátima Cano Cano^1^, Elena Lozano Soto^1^, Carmen García Guijo^1^, Javier J. González Rosa^1^

^1^H. U. Puerta del Mar, 11009 Cádiz, España

**Objetivos**: Aunque las medidas de daño cerebral obtenidas mediante RM resultan informativas del estado clínico de pacientes con Esclerosis Múltiple Remitente-Recurrente (EMRR), el valor pronóstico de biomarcadores sanguíneos como los neurofilamentos ligeros (NfL) y la proteína ácida fibrilar glial (GFAP) todavía está por determinar. Nuestro objetivo fue caracterizar los procesos de daño cerebral durante las primeras fases de la enfermedad y determinar su valor predictivo sobre el declive cognitivo y la discapacidad.

**Material y Métodos**: Un total de 35 pacientes EMRR recién diagnosticados y 22 controles sanos realizaron una evaluación basal y otra longitudinal a los 3 años que incluyó una exploración clínica y neuropsicológica, una extracción sanguínea para la cuantificación de NfL/GFAP y un estudio de RM para la obtención de medidas de volumetría cerebral y de grosor cortical. Se emplearon modelos estadísticos para explorar diferencias entre grupos y de regresión múltiple para examinar el valor predictivo de las variables de interés.

**Resultados**: Un 49% de los pacientes desarrolló un empeoramiento cognitivo a los 3 años, acompañado por una atrofia subcortical y cortical acelerada en comparación con los pacientes cognitivamente estables. La reducción del tálamo, el adelgazamiento cortical frontal y el estado de ánimo predijeron el estado cognitivo, mientras que el grosor cortical global y los niveles de GFAP predijeron el grado de discapacidad al final del estudio.

**Conclusiones**: Los niveles séricos de GFAP, junto con medidas de neuroimagen de daño en sustancia gris, constituyen marcas tempranas y distintivas de la progresión del deterioro cognitivo y la discapacidad en la EMRR.

**CO-04.- Neurotoxicidad en Relación a la Terapia CAR-T: Experiencia en Nuestro Centro**

Irene Lorite Fuentes^1^, Sergio Navarro Conti^1^, Alba Rodríguez Martín^1^, María Josefa Álvarez Soria^1^, Paula Martínez Agredano^1^

^1^H. U. Reina Sofía, 14004 Córdoba, España

**Objetivos**: La inmunoterapia con células T con receptor de antígeno quimérico (CAR) es una terapia innovadora para el tratamiento del Linfoma B difuso de células grandes (LBDCG). El uso de esta terapia conlleva en un alto porcentaje de los casos la aparición de complicaciones graves tales como el síndrome de liberación de citoquinas (SLC) y síndrome de neurotoxicidad asociada a células inmunoefectoras (ICANS). El objetivo del presente estudio es el análisis de la incidencia y evolución del ICANS en nuestro centro.

**Material y Métodos**: Estudio descriptivo retrospectivo que recoge los datos de 5 pacientes con LBDCG en recaída o refractarios a tratamiento que han sido tratados hasta ahora con CAR-T en el servicio de Hematología de nuestro hospital. Las principales variables recogidas fueron la edad, sexo, tipo de CAR-T empleado, RM, EEG, reactantes de fase aguda (RFA), grado SLC e ICANS, administración de Tocilizumab y corticoterapia.

**Resultados**: De los 7 pacientes recogidos en nuestro estudio todos ellos presentaron SLC, de los cuales el 60% desarrollaron SLC grado 1 y el 40% restante grado 3. En todos los casos de SLC se produjo un aumento de reactantes de fase aguda (IL-6, proteína C reactiva, ferritina). La incidencia de ICANS fue del 60%, de los cuales el 75% recibió terapia corticosteroidea por presentar signos clínicos de neurotoxicidad. Tras el uso de corticoterapia todos presentaron mejoría progresiva de la clínica neurológica.

**Conclusión**: El manejo y tratamiento precoz del ICANS asociado a infusión con CAR-T resulta determinante para la evolución favorable de estos pacientes.

**CO-05.- Similitudes y Diferencias en el Estatus Neurológico y su Asociación con Biomarcadores de Neurodegeneración Entre Pacientes con Diabetes Mellitus Tipo 1 y Esclerosis Múltiple**

Fátima Cano Cano^1^, Álvaro J. Cruz-Gómez^1^, Manuel Aguilar-Diosdado^1^, Ana I. Arroba^1^, Lucía Forero^1^, Javier J. González-Rosa^1^

^1^H. U. Puerta del Mar, 11009 Cádiz, España

**Objetivos**: A pesar de las diferencias clínicas de Esclerosis Múltiple (EM) y la diabetes mellitus tipo 1 (DMT1), nuevas evidencias apuntan a la existencia ciertas características comunes a nivel genético e inmunológico, aunque la comparación del estatus neurológico de ambas etiologías ha sido escasamente estudiada.

**Métodos**: Se reclutaron 18 pacientes con DMT1 y 35 con EM remitente-recurrente (EMRR) con <4 años de evolución, además de 23 controles sanos (CS). En estos sujetos se realizó: (i) exploración clínica y neuropsicológica, (ii) OCT, (iii) RM estructural cerebral, y (iv) concentración de NfL y GFAP en suero. Además, se analizaron los puntos (ii) y (iv) en otro grupo de 12 pacientes con DMT1 de >4 años de evolución.

**Resultados**: Los pacientes con EMRR mostraron numerosas alteraciones relacionadas con la presencia de déficits cognitivos y de cambios a nivel de OCT y de atrofia cerebral regional, mientras que los pacientes con DMT1 mostraron un estatus clínico similar a los CS en términos de ausencia de alteraciones neurológicas, cognitivas y radiológicas. Aunque los niveles de NfL y GFAP no parecieron diferenciar significativamente ambas patologías en estadios tempranos, los niveles de NfL y las alteraciones OCT se incrementaron en pacientes DMT1 con más años de evolución.

**Conclusiones**: Si bien la evidencia sugiere que la EM posee una mayor progresión hacia la neurodegeneración que la DMT1 desde estadíos tempranos, nuestros resultados apuntan hacia un aumento de las alteraciones neurológicas en la DMT1 en fases más tardías, siendo necesarios más estudios que exploren esta posibilidad.

**CO-06.- Efectos de la Estimulación Subtalámica Sobre la Integridad de la Actividad Cerebral Espontánea en Pacientes con Enfermedad de Parkinson**

Constantino Méndez Bértolo^1^, Florencia Sanmartino^1^, Raúl Rashid López^1^, Lijandy Jiménez Armas^1^, Diego Mateos^1^, Javier J. González Rosa^1^

^1^H. U. Puerta del Mar, 11009 Cádiz, España

**Objetivos**: Para comprender mejor los mecanismos de la estimulación cerebral profunda del núcleo subtalámico (STN-DBS), este estudio pretendió investigar el efecto de la estimulación sobre la actividad cerebral espontánea en las redes cerebrales a gran escala de pacientes con enfermedad de Parkinson (PEP).

**Métodos**: Se registró la actividad espontánea EEG en estado de reposo durante ojos abiertos (OA) y ojos cerrados (OC) en 14 PEP con tratamiento crónico de STN-DBS en condiciones Off-medicación y en estado DBS On/Off, y en 14 controles sanos (CS). Se estudiaron los cambios de la potencia espectral relativa y la variabilidad (entropía) de la actividad EEG en las bandas theta, alpha, low-beta y high-beta mediante estadística no-paramétrica con corrección por clusters.

**Resultados**: Respecto a los CS, los PEP mostraron un aumento en la potencia de theta y un decremento en low-beta. Durante el estado DBS-On se redujo la actividad theta patológica (con OC) y aumentó la potencia en low-beta (con OA). Asimismo, durante DBS-On la entropía aumentó en la banda theta en ambos estados de reposo e incrementó la actividad en la banda alpha durante OA.

**Conclusiones**: En ausencia de medicación dopaminérgica, la STN-DBS se asoció con: (i) una reducción de la actividad espectral EEG en frecuencias bajas y un aumentó las de frecuencias rápidas, (ii) una modificación de la complejidad de la actividad EEG en theta. Nuestros hallazgos sugieren que la potencia espectral y la complejidad de la señal cerebral varían dependiendo del estado de activación neural de los PEP bajo tratamiento con STN-DBS.

**CO-07.- Análisis de Factores de Riesgo y Recurrencia del Ictus Isquémico y sus Subtipos: Un Estudio Retrospectivo de dos Décadas**

Germán Antonio Escobar Rodríguez^1^, Alfredo Palomino García^1^, José Ramón Torres Martín^1^, Carlota Villar Rodríguez^1^, Auxiliadora Caballero García^1^, María Dolores Jiménez Hernández^1^

^1^H. U. Virgen del Rocío, 41013 Sevilla, España

**Objetivo**: El objetivo del estudio es caracterizar los factores condicionantes del riesgo de ictus isquémico y sus subtipos, incluida su recurrencia. La población a analizar es la de un hospital de tercer nivel y en un periodo de dos décadas.

**Material y Métodos**: Estudio es descriptivo, longitudinal y retrospectivo, basado en 57.873 informes clínicos de alta (IAHCE) de cualquier episodio clínico de 11.958 pacientes que presentan ictus entre 2000 y 2021. Muestreo aleatorio estratificado y técnicas de procesamiento del lenguaje natural (PLN) para identificar los ictus. Posterior identificación de 401 IAHCE de ictus isquémico y clasificación por subtipos: cardioembólico, aterotrombótico, ESUS, lacunar, indeterminado y otros. Análisis y cruce de los resultados con los factores de riesgo cardiovascular (FRCV). Se utilizó Python y Visual Studio Code para el análisis.

**Resultados**: Se identificaron 42.398 registros de ictus isquémico en 8327 pacientes. La hipertensión arterial fue el FRVC más prevalente (55,9% aterotrombótico, 52,5% cardioembólico). Los FRCV más importantes fueron diabetes, dislipemia y obesidad. La recurrencia de ictus 2,4%, menor que la reportada en la literatura. Los subtipos más frecuentes: cardioembólico y aterotrombótico. Se observó una asociación significativa entre factores de riesgo como hipertensión, tabaquismo y enfermedades metabólicas con los diferentes subtipos de ictus.

**Conclusiones**: Factores como el control de la hipertensión, la diabetes y la dislipemia son probablemente cruciales para la prevención de episodios recurrentes. La implementación de sistemas de información digital y técnicas de PLN son herramientas valiosas para dar soporte, contribuyendo a una mejor comprensión y manejo del ictus isquémico.

**CO-08.- Efectos de la Estimulación Cerebral Profunda Sobre Biomarcadores Séricos de Neurodegeneración y Neuroplasticidad en Pacientes con Enfermedad de Parkinson**

Florencia Sanmartino^1^, Fátima Cano Cano^1^, Raúl Rashid López^1^, Álvaro J. Cruz Gómez^1^, Raúl Espinosa Rosso^1^, Javier J. González Rosa^1^

^1^H. U. Puerta del Mar, 11009 Cádiz, España

**Objetivos**: Investigar el efecto de la estimulación cerebral profunda (ECP) en la enfermedad de Parkinson (EP) sobre biomarcadores séricos asociados con el daño neuroaxonal, la reactividad astrocitaria y la plasticidad neuronal.

**Métodos**: Se reclutaron 4 grupos de participantes: (i) 15 pacientes con EP seleccionados para recibir el tratamiento de ECP, evaluados antes, a la semana, al mes y al año tras la ECP; (ii) 15 pacientes con EP en tratamiento crónico de ECP; (iii) 17 pacientes con EP estables bajo tratamiento farmacológico; (iv) 17 controles sanos (CS). En todos los participantes se cuantificaron los niveles séricos de la cadena ligera de neurofilamentos (sNfL), la proteína ácida fibrilar glial (sGFAP) y el factor neurotrófico derivado del cerebro (sBDNF) mediante la técnica Simoa.

**Resultados**: En las primeras semanas posoperatorias observamos un aumento transitorio de sNfL y sGFAP, así también como una disminución temporal de sBDNF. Al año de seguimiento, las concentraciones de los biomarcadores estudiados retornaron a niveles similares a los basales. Además, los pacientes con EP presentaban concentraciones de sNfL significativamente más elevadas que los CS. Ninguno de los biomarcadores estudiados demostró tener la capacidad de discriminar entre los diferentes subgrupos de EP.

**Conclusiones**: Nuestros hallazgos confirman que la ECP no promueve la neurodegeneración, aunque la cirugía de ECP induzca procesos transitorios proinflamatorios y de daño neuronal. Los NfL pueden ayudar a rastrear la gravedad y la progresión de la enfermedad, aunque sus inconsistencias con la mejoría clínica cuestionan su validez hasta el momento.

## Comunicaciones Poster

**PO-01.- Quejas Cognitivas en Pacientes con Trasplante Alogénico de Progenitores Hematopoyéticos: A Propósito de un Caso, con Revisión de la Literatura**

Luque Ambrosiani Antonio Cristóbal^1^, Villagrán Sancho Diego^1^, Hernández Chamorro Francisco José^1^, Palomino García Alfredo^1^, Hernández Ramos Francisco José^1^

^1^H. U. Virgen del Rocío, 41013 Sevilla, España

**Objetivos**: Las quejas cognitivas en los pacientes que reciben un trasplante alogénico de progenitores hematopoyético (alo-TPH) son frecuentes, identificándose hasta en un 50% de los casos, siendo incapacitantes en más de un 25%. Presentamos un varón con alo-TPH por síndrome mielodisplásico que consulta por quejas cognitivas, dos años después de la inducción del trasplante.

**Material y Métodos**: Descripción de un caso clínico y revisión bibliográfica de publicaciones recientes sobre las quejas cognitivas en pacientes con alo-TPH.

**Resultados**: Varón de 59 años con antecedente relevante de síndrome mielodisplásico conocido desde 2013, refractario, precisando alo-TPH con inducción en 2021, sin complicaciones sistémicas posteriores, en tratamiento inmunosupresor crónico. Comenzó a finales de 2022 con olvidos de asuntos cotidianos, repetitivo en el día a día, con dificultad para recordar hechos recientes o tareas que tenía que realizar. Se interpretó inicialmente como posible deterioro cognitivo, por lo que se remitió a Neurología. En valoración ambulatoria de mediados de 2023, comentó mejoría sintomática, tras comenzar con antidepresivo y ansiolítico al identificarse crisis de ansiedad. Había vuelto al trabajo, sin fallos en el rendimiento en este y ya sin olvidos de hechos cotidianos. Se realizó RM craneal y test neuropsicológico, sin hallazgos destacables. Desde entonces, sigue activo laboralmente.

**Conclusiones**: Los pacientes con alo-TPH pueden presentar quejas cognitivas por múltiples causas, entre las que debemos descartar complicaciones sistémicas, por su gravedad. Pese a ello, en la mayoría de los pacientes la causa atribuible suele estar más en relación con ansiedad, depresión y/o insomnio, como ocurrió en nuestro caso.

**PO-02.- Perfil Cognitivo y Marcadores de Atrofia Cerebral en Pacientes con Enfermedad de Alzheimer de Inicio Precoz**

Mónica García Corrales^1^, Álvaro J. Cruz-Gómez^1^, Ana Gómez Roldós^1^, Luis Lobato^1^, Guillermo Rubio Esteban^1^, Javier J. González Rosa^1^

^1^H. U. Puerta del Mar, 11009 Cádiz, España

**Objetivos**: Este estudio tuvo como objetivo investigar el fenotipo cognitivo de pacientes con enfermedad de Alzheimer de Inicio Precoz (EAIP) en etapa de predemencia, así como las pruebas neuropsicológicas más sensibles para detectar sutiles déficits cognitivos. Además, se exploraron aquellos parámetros de integridad estructural cerebral que estaban más estrechamente relacionados con el estado cognitivo de los pacientes con EAIP.

**Material y Métodos**: Veintiséis participantes, incluidos 13 pacientes con diagnóstico de EAIP y 13 controles sanos (CS) emparejados en edad, sexo y nivel educativo, completaron una exhaustiva evaluación neuropsicológica. Los pacientes también llevaron a cabo una resonancia magnética estructural para cuantificar la cantidad de atrofia cerebral global y regional y el grosor cortical. El rendimiento cognitivo se comparó entre ambos grupos y en los pacientes se correlacionó con las medidas de volumetría cerebral.

**Resultados**: Los pacientes con EAIP mostraron un rendimiento cognitivo significativamente inferior al de los CS, el cual especialmente notable en dominios cognitivos relacionados con el lenguaje y la función visuoespacial. Las pruebas Mini-Linguistic State Examination, Test del Reloj y Stroop fueron las más sensibles para identificar déficits neuropsicológicos en estos pacientes. El deterioro cognitivo se asoció, además, con incrementos claros en la atrofia cerebral que implicaban ensanchamientos del tercer ventrículo y de los ventrículos laterales.

**Conclusiones**: Estos hallazgos respaldan la idea de que los déficits en el lenguaje, la velocidad de procesamiento y la función visoespacial pueden representar un sello cognitivo distintivo para la detección temprana y precisa de la EAIP.

**PO-03.- Características Conductuales y Neurales de la Afectación de la Memoria de Trabajo en Pacientes con Esclerosis Múltiple de Reciente Diagnóstico**

Esteban Sarrias Arrabal^1^, Álvaro J. Cruz Gómez^1^, Florencia Sanmartino^1^, Elena Lozano Soto^1^, Lucía Forero^1^, Javier J. González Rosa^1^

^1^H. U. Puerta del Mar, 11009 Cádiz, España

**Objetivos**: Se pretendió investigar si la afectación de diferentes subprocesos de la memoria de trabajo (MT) es un rasgo unitario en pacientes en fases tempranas de la Esclerosis Múltiple (EM), así como los correlatos cerebrales que sustentan la presencia de estos déficits.

**Material y Métodos**: 31 pacientes con diagnóstico reciente de EMRR fueron divididos en función de la afectación (MA) o preservación (MP) de la MT visuoverbal (tarea de Sternberg), y se compararon con un grupo de 18 controles sanos (CS). La actividad cortical involucrada en los subprocesos de MT fue analizada mediante la dinámica temporal del EEG y de sus generadores neurales, lo que se relacionó con medidas regionales de volumetría cerebral y grosor cortical.

**Resultados**: El grupo de pacientes EM-MP mostró un incremento de actividad neural en regiones frontales con respecto a los CS, mientras que los pacientes EM-MA manifestaron alteraciones funcionales en la fase consolidación de la MT (menor amplitud del ERP-P300) y un incremento de la actividad cerebral en regiones frontales y una disminución en regiones parietales. Además, este hallazgo en los pacientes EM-MA se acompañó de una reducción bilateral del volumen talámico y de un menor grosor cortical en áreas parietales derechas.

**Conclusiones**: La afectación distintiva de la MT visuoverbal y la desregulación funcional y estructural en redes tálamo-fronto-parietales en gran parte de los pacientes EM de reciente diagnóstico y baja discapacidad física apoyan el uso de estos biomarcadores como sellos distintivos que permitan discriminar entre fenotipos de EMRR desde estadios tempranos de la enfermedad.

**PO-04.- La Depresión en Enfermos de Parkinson se Relaciona con la Asimetría Interestriatal del Transportador de Dopamina**

Emilio Fernández Espejo^1^, Ana Santurtun^1^, Ángel Martin de Pablos^1^

^1^Laboratorio de Medicina Regenerativa de Málaga, 29015 Málaga, España

La dopamina y el transportador de dopamina (DAT) en los ganglios basales juegan un papel en la depresión. La enfermedad de Parkinson (EP) podría ser un buen modelo para estudiar la depresión de base dopaminérgica. El estudio se realizó entre 2010 y 2020, y se evaluaron 124 pacientes con EP (74 depresivos, 50 sin depresión) con tomografía computarizada de fotón único con I123-Ioflupano. Se valoró la ratio de fijación específica (Specific binding ratio o SBR), la asimetría interhemisférica del SBR, y la relación con variables motoras. Los resultados indican que la depresión es altamente prevalente en los enfermos de Parkinson (59,6%). El análisis de regresión logística multivariante ajustada con factores de riesgo de EP indica que la lateralización de la señal DAT, la duración de la enfermedad y la gravedad de los síntomas motores son predictores de EP. La fórmula logística de probabilidad de sufrir depresión es [log(P/1-P) = –11,452 + (33,553 × lateralización de DAT) + (0,341 × duración) + (0,162 × gravedad motora)]. El valor P obtenido con dicha fórmula es muy bueno para detectar depresión en los pacientes, con un valor de corte de 0,844, de alta precisión (0,969), sensibilidad (86,5%), y especificidad (92%). Los resultados podrían ser de utilidad para valorar la depresión en la EP.

**PO-05.- Cefaleas Primarias Relacionadas con la Menstruación ¿Es la Cefalea Igual Dentro y Fuera de la Menstruación?**

Pablo Olea Rodríguez^1^, María Luisa Ruiz Franco^1^, María del Mar Martínez Salmerón^1^, Patricia Perea Justicia^1^, Josefina Martínez Simón^1^, Antonio Arjona Padillo^1^

^1^H. U. Torrecárdenas, 04009 Almería, España

**Objetivos**: De acuerdo con la Tercera Edición de la Clasificación Internacional de Cefaleas-2018 (ICHD-III), la migraña menstrual tiene lugar entre los días –2 a +3 de la menstruación y existen dos tipos: migraña menstrual pura y migraña relacionada con la menstruación. Existe además evidencia clínica de otras cefaleas primarias relacionadas con la menstruación (CRM). En ellas no se ha estudiado si el tipo de cefalea es el mismo dentro (CDM) y fuera (CFM) de la menstruación. Nuestro objetivo es estudiar en una muestra de CRM estas posibles diferencias.

**Material y Métodos**: Realizamos un análisis post-hoc de un estudio observacional, transversal y prospectivo que incluyó 60 mujeres con CRM identificadas mediante un cuestionario validado y basado en la ICHD-III. De acuerdo con estos criterios diagnósticos, se estableció el diagnóstico de la CDM, si esta era igual a la CFM y, en caso de discordancia, también el tipo de CFM.

**Resultados**: De las 60 mujeres, 45 CDM migraña, 14 presentaron CDM tipo tensión y 1 CDM fue excluida por inclasificable. Las CDM y CFM fueron similares en 56 sujetos y distintas en 3 casos, todas con CDM migraña y CFM tipo tensión.

**Conclusiones**: En la mayoría de los casos la CDM y CFM son idénticas, pero pueden coexistir dos tipos de cefaleas primarias en una misma mujer. Es preciso realizar una anamnesis por separado para diferenciar la CDM y CFM. En nuestra serie, los fenotipos sugieren que se tratan de migrañas menstruales con crisis más leves fuera de la menstruación por influencia estrogénica.

**PO-06.- Realidad Virtual y Lenguaje en EP**

Jesús Paredes Duarte^1^, del Carmen Ayora Esteban^1^

^1^Instituto de Lingüística Aplicada-Universidad de Cádiz, 11002 Cádiz, España

**Objetivos**: Se realizó una investigación con un software de realidad virtual con el fin de mejorar desde el punto de vista motor y verbal a enfermos de párkinson. Se trabajaron fundamentalmente actividades de la vida diaria que pudieran, a su vez, incidir en la autonomía del paciente.

El objetivo principal del trabajo fue demostrar que la realidad virtual servía para rehabilitar el movimiento y, por tanto, el lenguaje en las personas afectadas de párkinson.

**Material y Métodos**: Se estudiaron 25 pacientes de párkinson con una escala de motricidad (UPDRS) de bajo índice, todos se sometieron a una evaluación del lenguaje inicial, a otra final y la mitad de ellos, a la realidad virtual. Para la evaluación del lenguaje recurrimos a test clásicos de evaluación como el test de Boston, el RIENAL, el Token test y el CEG y para la realidad virtual utilizamos el software Virtual Rehab de la empresa EVOLV.

**Resultados y Conclusiones**: En los contextos trabajados se observó una ligera mejora que podría ser más acuciada si se alargara en el tiempo el uso del citado software. Nos encontramos con mayores datos cualitativos que cuantitativos debido a la escasa muestra de participantes. Para trabajos posteriores, esta muestra debe ser ampliada y nos ofrecerá resultados más rigurosos.

**PO-07.- Una Revisión Sistemática Sobre la Utilidad de la Estimulación Magnética Transcraneal Como Tratamiento Para la Fatiga en Pacientes con Esclerosis Múltiple**

Álvaro González Moraleda^1^, Esteban Sarrias Arrabal^1^, Paloma Macías García^1^, Álvaro J. Cruz Gómez^1^, Lucía Forero^1^, Javier J. González Rosa^1^

^1^H. U. Puerta del Mar, 11009 Cádiz, España

**Objetivos**: La fatiga es considerada un desafío terapéutico y uno de los síntomas más discapacitantes en los pacientes con esclerosis múltiple (EM). Aunque la estimulación magnética transcraneal repetitiva (rTMS) ha demostrado gran eficacia clínica en el tratamiento de la fatiga o la depresión en numerosas condiciones neurológicas y psiquiátricas, su empleo en la EM es aún muy limitado. Este estudio llevó a cabo una revisión exhaustiva del uso de la rTMS y su eficacia terapéutica como modalidad de tratamiento para la fatiga en EM.

**Material y Métodos**: Se realizó una búsqueda en las bases de datos Pubmed/Web of Science hasta febrero de 2024 para identificar artículos que incluían ensayos clínicos controlados aleatorizados (RTC) de intervenciones con rTMS para tratar la fatiga como resultado principal o secundario en la EM.

**Resultados**: Un total de 5 RCT fueron finalmente seleccionados en esta revisión, los cuales englobaron a un total de 157 pacientes de RRMS, SPMS y PRMS. La aplicación de rTMS y otras de sus modalidades (deep TMS, iTBS) sobre áreas como el córtex prefrontal, la corteza motora o el cerebelo mostró una reducción significativa de la fatiga en comparación con la estimulación placebo. La combinación simultánea de rTMS y otras terapias resultó en una mejoría de la fatiga aún mayor.

**Conclusiones**: La rTMS podría proporcionar beneficios significativos a través de la reducción de la sintomatología asociada a la fatiga en pacientes con EM, aunque se necesitan más estudios para definir mejor protocolos estandarizados y parámetros de aplicación de rTMS en esta población.

**PO-08.- Bilingüismo y EP: Estudio Experimental con Verbos en el Marco del Modelo Declarativo-Procedimental con Hablantes Monolingües (Castellano) y Bilingües (Castellano-Catalán)**

Paredes Duarte María Jesús^1^, Diéguez Vide Faustino^2^, Zaragoza Cortés Maite^2^

^1^Instituto de Lingüística Aplicada, Universidad de Cádiz, 11002 Cádiz, España

^2^Universidad de Barcelona, 08007 Barcelona, España

**Objetivos**: Entre los modelos de procesamiento del lenguaje, el declarativo-procedimental distingue entre sistemas que funcionan por reglas (memoria procedimental) y aquellos que dependen principalmente del almacenaje de información (memoria declarativa). Este modelo sugiere que los sistemas basados en reglas se verían afectados en la EP, ya que son procesados en parte por los ganglios basales.

El objetivo principal del trabajo ha sido comparar las alteraciones lingüísticas en el ámbito de la conjugación verbal de pacientes con EP bilingües y monolingües en relación con la validez del modelo declarativo-procedimental.

**Material y Métodos**: Se estudiaron 10 pacientes monolingües y 10 bilingües catalán-castellano con competencia alta en todas las lenguas y estadios iniciales de la enfermedad. Se tuvo también en cuenta la variable nivel sociocultural. Se utilizó un test de evaluación de conjugación de verbos regulares e irregulares para valorar el modelo declarativo-procedimental comentado, según el cual la memoria procedimental regiría los verbos regulares y la declarativa los irregulares.

**Resultados y Conclusiones**: Las alteraciones lingüísticas encontradas en este contexto que comparan a individuos bilingües y monolingües con EP inciden en la localización de la lengua materna y la lengua o las lenguas adquiridas en el cerebro y su posible deterioro causado por la enfermedad. De este modo, se destaca la importancia de considerar el historial lingüístico del paciente para mejorar la atención individualizada.

**PO-09.- Aceruloplasminemia, a Propósito de un Caso**

Lara Mauri Fábrega^1^, María Teresa Fábregas Ruano^1^, Ana Rodríguez Reguera^1^

^1^H. U. Jerez de la Frontera, 11407 Cádiz, España

**Introducción**: La aceruloplasminemia es una enfermedad genética con herencia autosómica recesiva. Pertenece al grupo de enfermedades por “neurodegeneración en relación a acúmulo de hierro cerebral”. Debuta en edades tardías con trastornos neuropsiquiátricos y/o extrapiramidales. Analíticamente se caracteriza por niveles de ferritina altos con anemia ferropénica y el patrón radiológico de depósitos de hierro es típico.

**Material y Métodos**: Varón de 68 años con historia de hiperferritinemia idiopática asociada a anemia ferropénica de más de diez años de evolución y con estudio genético de hemocromatosis negativo. Consultó por deterioro cognitivo leve, trastorno psicótico tardío y discreto parkinsonismo exacerbado por neurolépticos. En RM cerebral se observó un particular patrón de distribución de depósitos de hierro afectando a núcleo dentado, núcleo rojo, sustancia negra, globo pálido, putamen y tálamo, sin lesiones cavitarias. Ante dichos resultados, se solicitó estudio de metabolismo del cobre y estudio genético de aceruloplasminemia. Hermana en seguimiento en otro centro por proceso clínico-radiológico superponible.

**Resultados**: Se objetivó un cobre sérico bajo, una cupruria en rango de normalidad y niveles de ceruloplasmina prácticamente indetectables. El estudio sistémico fue negativo, salvo una DM2 en el contexto de síndrome metabólico. El estudio genético de aceruloplasminemia resultó positivo. Se instauró tratamiento con quelantes de hierro.

**Conclusiones**: Existen menos de 60 casos de aceruloplasminemia descritos en la literatura hasta la fecha, es una enfermedad de las denominadas “ultrarraras”. El correlato clínico-analítico-radiológico es un denominador constante y típico, por lo que es preciso considerar esta entidad ante casos con un patrón superponible a los referidos.

**PO-12.- Enfermedad por Anticuerpos IgLON5: La Dificultad Diagnóstica de una Patología Emergente**

María Begoña Gómez González^1^, Juan Girón Úbeda^1^, Ana Rodríguez Román^1^, Alicia Sánchez Refolio^1^, Miriam Sillero Sánchez^1^

^1^H. U. Puerto Real, 11510 Cádiz, España

**Objetivos**: Comunicación del proceso diagnóstico de la enfermedad por anticuerpos IgLON5en una paciente.

**Material y Métodos**: Mujer de 72 años hospitalizada en enero/febrero de 2023 en la Unidad de Cuidados Intensivos por estridor respiratorio grave con parada cardiorrespiratoria y posteriores fibrilación auricular y shock hemorrágico por úlceras anales; se detectó paresia de una cuerda vocal, y en la evaluación por Neurología, se sospechó una Atrofia Multisistémica, por síntomas de un año de evolución de disfonía (valorada por ORL en 2022: resolución de edema leve de cuerdas vocales), disfagia, ralentización motora progresiva, rigidez, temblor, inestabilidad, incontinencia urinaria y soliloquios nocturnos.

En la evaluación de Consultas de nuestro centro (marzo 2023), portaba traqueostoma; bajo tratamiento con levodopa, había mínimo hipertono del miembro inferior derecho, disfonía y ausencia de lentificación. Como Antecedentes, figuraban Hipertensión Arterial, Síndrome leve de Apnea Obstructiva del Sueño (2021) y cese de consumo tabáquico.

**Resultados**: Se solicitaron SPECT cerebral, sin patología nigroestriatal, y determinación de anticuerpos antiIgLON5, positivos. En enero de 2024, tras vacunarse frente a la Hepatitis B y realizar profilaxis antituberculosa, sé administra rituximab. La paciente permanece estable.

**Conclusiones**: Dentro de las nuevas enfermedades inmunológicas vinculadas a anticuerpos específicos, la enfermedad por anticuerpos IgLON5 constituye una entidad de difícil diagnóstico por su rareza y por la similitud semiológica con los parkinsonismos atípicos y otras enfermedades degenerativas de expresión predominantemente bulbar. En nuestro caso, la complicación grave del estridor aceleró el proceso diagnóstico de una patología cuya etiología puede permanecer incierta durante años.

**PO-13.- Coma Alfa Arreactivo Reversible en Paciente con Síndrome Antigangliósido Solapado**

Lara Mauri Fábrega^1^, Inmaculada González Nieto^1^, María Teresa Fábregas Ruano^1^, Ana Rodríguez Reguera^1^

^1^H. U. Jerez de la Frontera, 11407 Cádiz, España

**Objetivos**: Los síndromes antigangliósidos pueden expresarse solapadamente. El síndrome de Bickerstaff, se caracteriza por la triada oftalmoplejía, ataxia y alteración de la conciencia. El patrón electroencefalográfico consiste normalmente en un enlentecimiento difuso inespecífico. Presentamos el caso de un síndrome antigangliósido solapado (Miller-Fisher y Bickerstaff) con expresión de coma alfa arreactivo reversible.

**Material y Métodos**: Paciente de 59 años que ingresó por un síndrome de Miller-Fisher de evolución tórpida, requiriendo ingreso precoz en UCI. El estudio de anticuerpos antigangliósidos fue positivo para GQ1b, GT1a y GT1b. A la semana del ingreso y sin sedación 72 horas, sufrió deterioro neurológico con signos de disfunción de diencefálica severa (Glasgow 3 y ausencia de reflejos troncoencefálicos). Se realizó TC con angio-TC, PL y EEG urgentes y RM preferente.

**Resultados**: El TC y la PL resultaron normales. El EEG mostró un patrón homogéneo de frecuencias alfa no reactivo a estímulos, “coma alfa arreactivo”. La neuroimagen RM resultó normal. Se consideró un cuadro solapado manteniéndose actitud expectante. El cuadro central revirtió en una semana, sin secuelas cognitivas siendo las secuelas periféricas severas.

**Conclusiones**: El ritmo alfa se genera por defecto en el tálamo, siendo regulado por la sustancia reticular activadora ascendente. Cuando existe una lesión funcional o estructural de esta última, puede objetivarse un patrón de ritmos alfa difusos (coma alfa) debido a la toma de control del marcapasos talámico. El coma alfa arreactivo no ha sido previamente descrito en el síndrome de Bickerstaff, es causa de coma alfa arreactivo reversible, y es preciso considerarlo en este espectro de síndromes autoinmunes.

**PO-14.- Manifestación Cerebrovascular del Shock Anafiláctico**

Cecilia Coronado Puerto^1^, Julio Ruiz García^1^, Alejandro Molinero Marcos^1^, María Acosta de los Reyes^1^, Irene Serrano García^1^, Manuel García Ortiz^1^

^1^H. U. Puerta del Mar, 11009 Cádiz, España

**Objetivos**: Exposición de un caso clínico de un paciente que sufre un ictus isquémico de causa infrecuente.

**Material y Métodos**: Se presenta un varón de 40 años que tras sufrir numerosas picaduras de insectos, seguidas de una reacción anafiláctica presenta una focalidad neurológica hemisférica derecha, con una puntuación en la escala NIHSS de 15 puntos. Analítica con hemograma, bioquímica y coagulación así como TAC de cráneo simple y AngioTC urgentes resultan sin hallazgos.

**Resultados**: Durante su ingreso se lleva a cabo diagnostico etiológico con ecocardiografía con test de burbuja, holter 24 h y marcadores analíticos de hipercoagulabilidad dentro de la normalidad. Se realiza RNM de cráneo con secuencia de difusión que muestra múltiples infartos hemisféricos derechos en territorios de ACM y ACP sin objetivar ateromatosis carotidea. Tras una revisión bibliográfica se encuentran referencias de patología cerebrovascular asociada a anafilaxia con un mecanismo similar al que ocurre en el síndrome de kounis. El síndrome de kounis es una manifestación coronaria de la anafilaxia donde se produce una vasoconstricción tanto en coronarias sanas como con ateroesclerosis.

**Conclusiones**: El síndrome de kounis es una manifestación de la anafilaxia poco frecuente y puede tener relación con el mecanismo de los infartos cerebrales tras sufrir una anafilaxia.

**PO-16.- Síndrome de Clippers, a Propósito de un Caso**

Lara Mauri Fábrega^1^, María Teresa Fábregas Ruano^1^, Ana Rodríguez Reguera^1^

^1^H. U. Jerez de la Frontera, 11407 Cádiz, España

**Introducción**: El síndrome de CLIPPERS, de sus siglas en inglés, inflamación linfocítica crónica y perivascular pontina con respuesta a esteroides, se caracteriza: clínicamente, por sintomatología aguda de troncoencéfalo y cerebelo con buena respuesta a esteroides; radiológicamente, por lesiones hiperintensas <3 mm con realce homogéneo (patrón “sal y pimienta”) y; anatomo-patológicamente, por infiltrados perivasculares de linfocitos T-CD4+.

**Material y Métodos**: Paciente de 38 años que ingresó por diplopía, ataxia y disartria de un día de evolución. Se descartó patología vascular e infecciosa aguda, se realizó RM cerebral con contraste, se llevó a cabo amplio despistaje de causa sistémica, y se instauró tratamiento empírico con corticoides.

**Resultados**: En RM cerebral se objetivaron hiperintensidades puntiformes en corona radiada, tálamo, mesencéfalo y protuberancia que realzaban homogéneamente con gadolinio en “patrón en sal y pimienta”. No se filió causa sistémica del cuadro. La respuesta a corticoides fue buena, presentando recaída en relación a retirada precoz, por lo que se reinstauraron y retiraron de forma más paulatina. Se decidió tratamiento de mantenimiento con tocilizumab sin recaídas al año. No se realizó biopsia.

**Conclusiones**: El síndrome de CLIPPERS es una entidad rara. El patrón clínico-radiológico característico puede ser común a otras entidades, siendo necesario un amplio despistaje de secundariedad. La corticoterapia inicial. Ha de ser incisiva y su retirada lenta, debido al alto riesgo de recidiva. El tratamiento de mantenimiento con tocilizumab es anecdótico en la literatura, pero con buenos resultados, al igual que en nuestra paciente, por lo que habría que considerarlo como opción terapéutica.

**PO-17.- A “Gilliat-Sumner Hand”, Como Presentación de un Síndrome del Desfiladero Torácico**

Alicia Fernández Panadero^1^, Pablo Andrés García Núñez^1^, Andrés Hermosín Gómez^1^

^1^H. U. Virgen del Rocío, 41013 Sevilla, España

**Objetivos**: El síndrome del desfiladero torácico (SDT) neurogénico es una causa de plexopatía braquial compresiva infrecuente que debe considerarse dentro del diagnóstico diferencial de patologías del SNP para evitar su infra diagnóstico.

**Material y Métodos**: Mujer de 62 años, con debilidad progresiva de la mano derecha de 2 años de evolución, orientada inicialmente como patología articular, posteriormente valorada por Traumatología y diagnosticada de síndrome del túnel del carpo y neuropatía cubital derecha a nivel del codo, siendo intervenida. En la exploración destaca atrofia de APB, ADM e interóseos, hipoestesia en territorio cubital de la mano derecha, hiperreflexia generalizada, salvo estilo radial derecho abolido, y Hoffman izquierdo. Ante no mejoría y progresión clínica se replantea el caso.

**Resultados**: Se realiza RMN cráneo-cervical que descartó mielopatía y radiculopatía. En el estudio de conducción se aprecia afectación de los nervios mediano, cubital y cutáneo antebraquial medial, compatible con afectación del cordón medial del plexo braquial derecho. En TC Torácico-cervical se encuentra costilla cervical bilateral. En angioTC cérvico-torácico en abducción se objetiva estenosis dinámica vascular grave pero sin alteración estructural. Finalmente, se realiza el diagnostico de SDT neurógeno, no siendo candidata a tratamiento quirúrgico.

**Conclusiones**: El SDT es un diagnóstico infrecuente, a considerar ya que, en ocasiones, se acompaña de compresión de estructuras vasculares (arteria y vena subclavia) que requiere descompresión quirúrgica urgente. Se debe englobar dentro del diagnóstico diferencial de patologías neuromusculares como la amiotrofia monomiélica, ELA (si asocia piramidalismo, como en nuestro caso), radiculopatía cervical, síndrome Parsonage-Turner o neuropatías periféricas, entre otros.

**PO-18.- Un Brazo por Cuenta Propia: Un Caso Sobre Mano Alienígena**

Alexandra Rincón Valencia^1^, Ignacio Ruiz Salcedo^1^, Coral Conde Velasco^1^, Víctor Carmona Bravo^1^, Soledad Pérez Sánchez^1^, Joan Montaner Villalonga^1^

^1^H. U. Virgen Macarena, 41009 Sevilla, España

**Introducción**: El síndrome de la mano alienígena se caracteriza por movimientos involuntarios e incontrolables del miembro afecto, como “controlado por una fuerza externa”. Varias etologías implicadas: accidente cerebrovascular, esclerosis múltiple, Creutzfeld-Jakob o degeneración corticobasal, siendo la vascular la más común.

**Material y Métodos**: Varón de 51 años sin AP reseñables, presenta cuadro brusco de alteración del lenguaje y debilidad hemicuerpo izquierdo, con hemihipoestesia izquierda. Posteriormente, posturas anómalas involuntarias de mano y pie izquierdos (poner pie sobre la barandilla, agarrarse la ropa con un puño) que logra resolver voluntariamente. En TC de cráneo y Angio-TC se objetiva hemorragia lobar frontal derecha secundaria a MAV tipo fistula dural. Confirmación mediante arteriografía, donde se identifica trombosis del seno recto como origen fistuloso. EEG normal.

**Resultados**: Embolización de la fístula, observándose en neuroimagen de control fístula arterio-venosa del seno sagital superior residual, que se cierra. Como resultado, mejoría del déficit sensitivo-motor izquierdo.

**Conclusiones**: Nuestro paciente presentaba rasgos típicos de la variante frontal del síndrome (tanteo impulsivo, manipulación compulsiva de objetos y clínica frontal), por lesión de corteza prefrontal medial y área motora suplementaria. Otras variantes son la callosal, destacando el conflicto intermanual; y la variante posterior (temporo-parietal, occipital y/o talámica), con alienación y levitación de extremidad, ataxia sensorial y clínica parietal. El pronóstico varía según la etiología, siendo el inducido por causa vascular el mejor. Si el infarto es unilateral, la parte sana compensa la función deteriorada y reconstruye la conexión interhemisférica. Así sucedió con nuestro paciente, que presentó resolución clínica completa al alta.

**PO-19.- Ravulizumab y Miastenia Gravis Generalizada. Presentación del Primer Paciente Tratado en España**

García Ortiz Manuel^1^, Molinero Marcos Alejandro^1^, Acosta de los Reyes María^1^, Coronado Puerto Cecilia^1^, Serrano García Irene^1^, Fernández Navarro José^1^

^1^H. U. Puerta del Mar, 11009 Cádiz, España

**Objetivos**: Ravulizumab es un anticuerpo monoclonal IgG2 que actúa sobre C5, impidiendo su escisión en C5a (anafilotoxina) y C5b (CAM). El complemento es un conjunto de proteínas plasmáticas que dirigen la fagocitosis y lisis celular en la respuesta inmunitaria innata. La MG es una enfermedad autoinmune neuromuscular que se caracteriza por debilidad muscular fatigable. El 85% de los pacientes presentan autoanticuerpos ACHR+, ha quedado demostrado que estos anticuerpos inducen la escisión de C5 para formar un CAM que resulta en la destrucción de la membrana neuromuscular post-sináptica.

**Material y Métodos**: Varón de 63 años con AP: HTA, DLP. Debuta en 06/2022, con cuadro bulbar en forma de disfagia a líquidos y disnea a moderados esfuerzos, siendo diagnosticado en 07/2023 de MG IIb según clasificación MGFA con atcp. ACHR+. Durante el siguiente año necesita ingreso con colocación de SNG (MGFA IIIb) y tratamiento con inmunoglobulinas. Recibe tratamiento sintomático con piridostigmina 90 mg c/6 h e inmunosupresión con tacrolimus (fracaso de azatioprina), el cual no tolera por RAM. Acaba precisando tratamiento trimestral con inmunoglobulinas por crisis bulbares.

**Resultados**: Debido a fracaso o intolerancia de tratamientos inmunosupresores y crisis miasténicas recurrentes, comienza tratamiento con Ravulizumab. Previo al inicio del fármaco, puntuación en escala MG-ADL de 9 puntos. Tras infusión de dosis de mantenimiento, puntuación en MG-ADL de 2 puntos, con reducción de tratamiento sintomático a piridostigmina 60 mg c/8 h y franca mejoría clínica.

**Conclusiones**: Ravulizumab se presenta como una alternativa terapéutica en paciente con MG ACHR+ refractarios a tratamiento inmunosupresor convencional, con un mecanismo de acción específico contra su desencadenante fisiopatológico que puede disminuir el número de RAM indeseables de la inmunosupresión generalizada.

**PO-20.- Fenómeno de Carótida Evanescente, Estado Protrombótico en Relación a Cáncer de Mama**

Lara Mauri Fábrega^1^, Paula García Rodríguez^1^, María Teresa Fábregas Ruano^1^, Ana Rodríguez Reguera^1^

^1^H. U. Jerez de la Frontera, 11407 Cádiz, España

**Objetivos**: Los estados de hipercoagulabilidad en relación al cáncer de mama son bien conocidos, incrementando el riesgo de sufrir infartos isquémicos cerebrales hasta en un 5%. Asimismo, el letrozol incrementa el riesgo de sufrir cualquier tipo de isquemia arterial en un 14% frente a tamoxifeno.

**Material y Métodos**: Mujer de 80 años con historia de carcinoma lobulillar infiltrante tratado mediante tumorectomía hacía un año y radioterapia adyuvante. Realizaba tratamiento con letrozol. Sin evidencia de recaídas en sucesivos controles. Ingresó por afasia del despertar en relación ictus isquémico agudo por oclusión del segmento M2 de la arteria cerebral media izquierda (resto de árbol carotídeo permeable). Doce horas tras el inicio de los síntomas, sufrió un deterioro neurológico brusco a expensas de un síndrome deficitario completo de arteria cerebral media izquierda y puntuando 21 en escala NIHSS. No se habían detectado arritmias en telemetría. Se repitió un nuevo estudio cerebrovascular urgente.

**Resultados**: En la angio-tomografía se objetivó una trombosis completa del árbol carotídeo izquierdo, desde el origen de la arteria carótida común e incluyendo la arteria cerebral media izquierda en su totalidad. Clínicamente evolucionó rápidamente hacia ictus maligno falleciendo en cuestión de horas.

**Conclusiones**: El cáncer de mama, así como el tratamiento con inhibidores de la aromatasa, incrementan sustancialmente el riesgo de trombosis arterial. En esta paciente, sospechamos que ambas circunstancias confluyeron y puede que algún factor criptogénico adicional. Ante fenómenos trombóticos arteriales atípicos, es preciso considerar la presencia de un estado protrombótico sobreimpuesto.

**PO-21.- Polineuropatía por Anticuerpos Anti-MAG, a Propósito de un Caso**

Lara Mauri Fábrega^1^, Teresa Fábregas Ruano^1^, Ana Rodríguez Reguera^1^

^1^H. U. Jerez de la Frontera, 11407 Cádiz, España

**Introducción**: La polineuropatía autoanticuerpos anti-glicoproteína asociada a la mielina (anti-MAG), es una entidad rara. Suele asociarse a gammapatía monoclonal IgM, pudiéndola preceder en el tiempo.

**Material y Métodos**: Varón de 78 años, sano, con cuadro inestabilidad de tres años de evolución, sin otros trastornos sensitivos. En la exploración destacaban: ausencia de reflejos aquíleos, dificultad en el tándem y Romberg inestable. Las distintas modalidades sensitivas estaban preservadas. Se solicitó neurografía, analítica y anticuerpos anti-MAG.

**Resultados**: La neurografía evidenció una polineuropatía sensitiva, de fibra gruesa, axonal, y de distribución tetrapendicular. Analíticamente, destacar una Hb glicosilada de 6,5%, un pico monoclonal IgM-kappa y un índice de anticuerpos anti-MAG de 9,45 (N <1).

**Conclusiones**: La polineuropatía anti-MAG cursa con un patrón neurográfico inicialmente desmielinizante, susceptible de evolucionar hacia axonal debido a que la inflamación local mantenida daña estructuras colaterales. Es preciso no desestimar este diagnóstico ante una polineuropatía a filiar con patrón axonal.

**PO-22.- Síndrome de Persona Rígida de Probable Etiología Inmunomediada, a Propósito de un Caso**

Ana Campos Villegas^1^, Antonio Javier Guzmán Téllez^1^, Francisco Pinel Ríos^1^

^1^H. U. Virgen de la Victoria, 29010 Málaga, España

**Objetivos**: El síndrome de persona rígida (SPS) es un trastorno poco común que afecta principalmente a mujeres entre 20 y 50 años, caracterizado por rigidez y espasmos musculares. El espasmo muscular episódico precipitado por movimientos repentinos, ruido o malestar emocional es una característica sensible y específica del SPS, conocida como reflejo de sobresalto. Se cree que su etiología está relacionada con autoanticuerpos que afectan las vías GABAérgicas.

**Material y Métodos**: Descripción de un caso clínico.

**Resultados**: Mujer de 61 años con antecedentes personales de cáncer de mama y bocio multinodular tóxico. Acude a nuestro hospital para seguimiento de SPS. Se realizan pruebas de imagen sin hallazgos patológicos. Se objetivan anticuerpos Anti GAD en LCR con 19,853 U/mL y positivización de Ac AntiGAD 65 en suero. En el estudio electromiográfico se registra una actividad muscular continua en reposo consistente en potenciales de unidad motora indistinguible de los voluntarios, sin mejora con contracción de músculos antagonistas. A la exploración destacan reflejos de estiramiento muscular con aumento de área reflexógena global, Hoffman bilateral, marcha de puntillas con rigidez de ambos miembros y axial, con pasos cortos y aumento de base de sustentación. La paciente fue tratada con clonazepam, baclofeno e inmunoglubulinas sin respuesta satisfactoria. Se realizan 6 sesiones de plasmaféresis en nuestro centro con escasa mejora.

**Conclusión**: El SPS sigue siendo un trastorno poco comprendido tanto en su patogénesis, diagnóstico y tratamiento, con un pronóstico variable y limitante para los pacientes. Este caso clínico busca contribuir al conocimiento de esta enfermedad en la literatura científica.

**PO-23.- Manejo Urgente de la Oclusión de la Arteria Oftálmica Durante la Embolización de Fístulas Arteriovenosas Durales a Través de Ramas de la Arteria Meníngea Media: A Propósito de un Caso**

Antonio Cristóbal Luque Ambrosiani^1^, Marta Aguilar^1^, Manuel Medina Rodríguez^1^, Alejandro González^1^, Francisco Moniche^1^

^1^H. U. Virgen del Rocío, 41013 Sevilla, España

**Objetivos**: La oclusión de la arteria oftálmica (AO) es una complicación posible en el abordaje endovascular de fístulas arteriovenosas durales (FAVD) a través de la arteria meníngea media (AMM), debido a las anastomosis entre ambas arterias. Reportamos un caso de oclusión de AO en estas circunstancias, así como su manejo.

**Material y Métodos**: Descripción del caso clínico y revisión breve de la literatura incluyendo los artículos sobre la oclusión de AO en la realización de procedimientos endovasculares.

**Resultados**: Varón de 80 años con hallazgo incidental de FAVD tipo III de Cognard a vena cortical parietal parasagital derecha con drenaje al seno longitudinal superior y aportes arteriales desde AMM ipsilateral, que fue tratada mediante embolización desde rama de AMM mediante inyección de glue puro mezclado con tántalo, apreciándose migración accidental a AO derecha por la arteria falcina anterior. Se identificó midriasis derecha, por lo que se inició masaje ocular y se elevaron tensiones arteriales inmediatamente. Posteriormente, se realizó EcoDoppler en sala demostrando flujo anterógrado en AO. Tras finalizar el procedimiento, el paciente presentó pérdida visual, siendo sólo capaz de contar dedos con el OD, la cual mejoró durante el postoperatorio hasta retornar a su visión basal antes del alta.

**Conclusiones**: Las anastomosis entre AO y AMM deben ser tenidas en cuenta al realizar embolizaciones desde esta última arteria y así evitar migración del material de embolización, especialmente al aplicar presión elevada durante la inyección. El masaje ocular y la elevación de tensiones arteriales pueden ser útiles en el momento agudo.

**PO-24.- Catatonía Como Manifestación Clínica en Encefalitis Límbica: Presentación de un Caso y Revisión de la Literatura**

Luque Ambrosiani Antonio Cristóbal^1^, Hernández Chamorro Francisco José^1^, Villagrán Sancho Diego^1^, Hernández Ramos Francisco José^1^, Palomino García Alfredo^1^

^1^H. U. Virgen del Rocío, 41013 Sevilla, España

**Objetivos**: La catatonía es una de las manifestaciones neuropsiquiátricas que podemos encontrar en pacientes con encefalitis límbica. La identificación de esta y el estudio de sus precipitantes es fundamental, pues puede resultar muy incapacitante. Presentamos el caso de una mujer con encefalitis límbica seronegativa que presentó empeoramiento en forma de catatonía, en la evolución de su enfermedad.

**Material y Métodos**: Descripción de un caso clínico y revisión bibliográfica de publicaciones recientes sobre la catatonía en las encefalitis.

**Resultados**: Mujer de 59 años, sin antecedentes relevantes. Comienza en 2020 con trastorno conductual y déficit mnésico severos, diagnosticándose de encefalitis límbica tras estudio extenso, sin causa ni anticuerpo asociados. Se trató con corticoides, inmunoglobulinas y rituximab, con mejoría parcial, pudiendo vivir sola precisando mínima ayuda. En 2022, presenta deterioro acusado, apareciendo rigidez simétrica y posturas mantenidas de miembros, con obediencia automática. Ingresa con sospecha de nuevo brote de encefalitis y se realiza estudio sin hallazgos, sí apreciándose toma de quetiapina a dosis elevadas con aumento reciente de dosis. Se reducen dosis de quetiapina hasta suspenderla, con mejoría parcial al alta, identificándose esta como precipitante, con diagnóstico final de estado catatónico. Desde entonces, la paciente permanece institucionalizada, por incapacidad para manejo ambulatorio autónomo.

**Conclusiones**: La catatonía es una característica frecuente de los pacientes con encefalitis, especialmente por anti-NMDA, encontrándose hasta en un 70% de estos. Su diagnóstico es fundamental para corregir los precipitantes y evitar las complicaciones, como fue el caso de nuestra paciente, en la que se asoció al consumo de neurolépticos y mejoró con su retirada.

**PO-25.- Carbamazepina Como Tratamiento de los Trastornos Paroxísticos Motores: A Propósito de un Caso de Esclerosis Múltiple**

Ignacio Ruiz Salcedo^1^, Joaquín Arzalluz Luque^1^, Fernando Francisco Ortega Ortega^1^, Juan Luis Ruiz Peña^1^, Guillermo Navarro Mascarell^1^, Miriam Ben Yelun Inserser^1^, Julio Dotor García Soto^1^, Rocío López Ruiz^1^, Sara Eichau Madueño^1^

^1^H. U. Virgen Macarena, 41009 Sevilla, España

**Objetivos**: Los trastornos paroxísticos son síntomas típicos de corta duración que ocurren espontáneamente o desencadenados por movimientos/estímulos sensitivos. En la Esclerosis Múltiple Remitente Recurrente (EMRR) pueden suponer un brote o secuelas de brotes previos. Se presenta el caso de una paciente con EMRR y síntomas paroxísticos faciobraquiales motores con buena respuesta a Carbamazepina.

**Material y Métodos**: Mujer de 49 años con EMRR de alta actividad e inicio reciente de Ofatumumab. Último brote consistente en disartria, torpeza de miembro superior derecho y parestesias en miembros izquierdos, con nueva lesión captante en centro semioval izquierdo y recuperación completa con corticoides. Dos meses después, comienza con episodios paroxísticos de contracción involuntaria tónica de hemicara, cuello y brazo derechos, desencadenados por el movimiento y autolimitados en 10 segundos progresivamente.

**Resultados**: Se realizó una RM cráneo con gadolinio sin evidencia de actividad, analítica sin datos de infección ni elevación de reactantes de fase aguda y EEG vigilia durante el cual tuvo lugar un episodio sin traducción electroencefalográfica. Ante sospecha de discinesias paroxísticas cinesigénicas, se introdujo Carbamazepina con desaparición de los episodios paroxísticos sin recurrencia. Se realizó electro neuromiografía facial derecha tras la introducción de Carbamazepina sin hallazgos sugerentes de discinesia, distonía ni otros trastornos del movimiento.

**Conclusiones**: Las discinesias paroxísticas cinesigénicas pueden desencadenarse por múltiples causas, como lesiones desmielinizantes en vía piramidal. La Carbamazepina está descrita como tratamiento de fenómenos paroxístos en EM, incluidas las discinesias paroxísticas. Dada su potencial refractariedad, es importante detectarlos, descartar actividad de la EM y aplicar tratamiento, siendo la carbamazepina una de las opciones.

**PO-26.- “Mano Caída” Bilateral por Citomegalovirus**

Pablo Hernández Vitorique^1^, Marta Vicente Domínguez^1^, Minerva Mañez Sierra^1^, Virginia Delgado Gil^1^, Ester Morales García^1^

^1^H. U. Virgen de la Victoria, 29010 Málaga, España

**Introducción**: La paraplejía bilateral de las manos es una presentación atípica de mielitis transversa. Aunque generalmente se observa en mielitis isquémicas, en raras ocasiones puede ocurrir como debut de una afectación parainfecciosa.

**Material y Métodos**: Presentamos el caso de una paciente de 15 años que debutó con una mielitis transversa parainfecciosa por Citomegalovirus, manifestando “mano caída” bilateral.

**Resultados**: Mujer de 15 años presentó un episodio brusco de dolor dorsal y pérdida de fuerza simétrica en ambas manos, preservando la fuerza proximal en los miembros superiores. Como antecedente, la paciente mencionó una gastroenteritis semanas antes. En la exploración física, estaba afebril, sin cefalea ni signos meníngeos. El balance muscular mostró fuerza 5/5 en abducción de hombros y flexo-extensión de codos bilateralmente, con pérdida total de fuerza en todos los músculos de ambas manos y fuerza normal en miembros inferiores. Se detectó un nivel sensitivo en D6 y arreflexia generalizada, excepto en los reflejos aquíleos (+/++++). Una resonancia magnética con contraste de cráneo y columna reveló lesiones en el cordón medular cervical C4–C7, con afectación central y anterior, compatibles con mielitis transversa aguda. Los estudios en suero y líquido cefalorraquídeo fueron normales, exceptuando una serología IgM positiva para CMV en suero. La paciente fue tratada con corticoides y rehabilitación, mostrando una buena evolución clínica.

**Conclusiones**: Aunque la paraplejía distal de las manos es una presentación infrecuente de las mielitis transversas parainfecciosas, su aparición súbita debe incluirse en el diagnóstico diferencial de afectación medular para posibilitar un abordaje precoz.

**PO-27.- Norse de Causa Infrecuente: Estatus Superrefractario Como Forma de Presentación de Encefalopatía Posterior Reversible**

Mikel Salgado Irazábal^1^, Antonio Cristóbal Luque Ambrosiani^1^, Francisco José Hernández Chamorro^1^, Francisco Javier Gómez Fernández^1^

^1^H. U. Virgen del Rocío, 41013 Sevilla, España

**Objetivos**: El Estado Epiléptico Refractario de Aparición Reciente (NORSE por sus siglas en inglés) se define como una presentación clínica, en un paciente sin epilepsia activa o patología neurológica, que presenta un estado epiléptico refractario sin una causa estructural, tóxica o metabólica aguda. Esta tiene un amplio diagnóstico diferencial siendo las causas identificadas más comunes inmune o infecciosa. Presentamos el caso de una mujer que comienza con un estatus super refractario secundario diagnosticándose posteriormente una encefalopatía posterior reversible.

**Material y Métodos**: Descripción de un caso clínico y revisión bibliográfica de publicaciones recientes sobre NORSE.

**Resultados**: Mujer de 56 años con antecedentes de Hipertensión, Dislipemia y Cardiopatía hipertensiva. Comenzó de manera súbita con automatismos orofaciales y desconexión del medio. Posteriormente la paciente comienza con múltiples crisis tónico-clónicas generalizadas siendo trasladada a hospital donde se realiza TC craneal sin hallazgos. Posteriormente la paciente continúa con crisis generalizadas sin respuesta a benzodiacepinas ni FAEs por lo que ingresa en la UCI iniciándose sedación. Se realiza nuevo TC, AngioTC y punción lumbar sin alteraciones. Así mismo se realiza EEG con disfunción cerebral, pero sin actividad epiléptica. Tras ajuste de antiepilépticos se consigue estabilización, permitiendo realizar RM cráneo que muestra datos compatibles con encefalopatía posterior reversible. Tras control de tensión arterial presenta una buena evolución pasando a planta de hospitalización sin nuevas crisis siendo posteriormente dada de alta.

**Conclusiones**: En el NORSE es vital un óptimo manejo en fase aguda, siendo posteriormente clave el diagnóstico etiológico que permita una terapia dirigida y así reducir su morbimortalidad.

**PO-28.- Respuesta Antimioclónica Mantenida con Perampanel en Enfermedad de Creutzfeldt-Jakob, a Propósito de un Caso**

Sergio Carlos Navarro Conti^1^, José Carlos Estévez María^1^, Irene Lorite Fuentes^1^

^1^H. U. Reina Sofia, 14004 Córdoba, España

**Objetivos**: La enfermedad de Creutzfeldt-Jakob se trata de una enfermedad priónica, donde la proteína patogénica puede tener un origen esporádico, genético o adquirido. La clínica cardinal consiste en afectación de funciones superiores, mioclonías ante sobresaltos, así como signos de piramidalismo y extrapiramidalismo. Es universalmente mortal ya que no tiene tratamiento eficaz.

**Material y Métodos**: Presentamos una mujer de 55 años valorada en 2019 por fallos en la escritura y en el cálculo, así como un deterioro en el lenguaje expresivo de un año de evolución. Su marido relataba sobresaltos durante la noche. A la exploración la paciente presentaba disfasia mixta de predominio motor, agrafia, apraxia de la escritura e ideomotora, apraxia mielocinética y estereotipias en mano derecha, así como mioclonías axiales y de miembros a la palmada. Hiperreflexia generalizada. Por mioclonías, se pauta ácido valproico y, ante ausencia de respuesta, se pauta Perampanel 5 mg/24 h. La paciente actualmente se encuentra mutista, con apraxia global, distonía e inquietud motora, sin mioclonías.

**Resultados**: En RM craneal se aprecia restricción a difusión en circunvoluciones posteriores, caudado y lenticular izquierdo, ínsula y frontal izquierdo. En EEG se objetiva lentificación difusa con ondas trifásicas. LCR normal, con antineuronales, proteína 14-3-3 y RT-QUIC negativos. Onconeuronales en sangre negativo. Estudio genético del gen PrPP y de epilepsia mioclónica progresiva negativa.

**Conclusiones**: Es de destacar el papel del Perampanel como antimioclónico, ya que desde la presunción diagnostica, se pauta dicho fármaco, consiguiendo el control monofásico de mioclónicas hasta el momento actual, a pesar de la evolución cognitiva y el curso tórpido de la enfermedad.

**PO-29.- Síndrome del Desfiladero Torácico Superior por Costilla Cervical: A Propósito de un Caso de Debilidad Muscular Progresiva en Paciente Joven**

Ignacio Ruiz Salcedo^1^, Eva María Martínez Fernández^1^, Coral Conde Velasco^1^, Alexandra Rincón Valencia^1^

^1^H. U. Virgen Macarena, 41009 Sevilla, España

**Objetivos**: El síndrome del desfiladero torácico superior está descrito en pacientes jóvenes con cuadros de dolor, parestesias y amiotrofia de cara medial de brazo, antebrazo y 4° y 5° dedos. Ocurre por mecanismos de compresión, angulación o estiramiento del tronco inferior del plexo braquial. Se presenta el caso de una paciente con debilidad progresiva de miembro superior derecho (MSD) sin mejoría tras resección de costilla cervical.

**Material y Métodos**: Mujer de 47 años con antecedentes de lupus eritematoso sistémico, que con 22 años comienza con pérdida de fuerza progresiva en mano derecha. Niega dolor. No alteración en miembro contralateral. En la exploración, debilidad severa de musculatura dependiente de mediano y cubital. Hipoalgesia en zona medial de antebrazo y analgesia de zona cubital de mano. Reflejos en MSD disminuidos y amiotrofia de la extremidad.

**Resultados**: Se realizó una electro neuromiografía de MSD con ausencia de respuesta motora y sensitiva de nervios cubital y mediano. Se realizó tenotomía de escalenos sin mejoría de la sintomatología. En radiografía de tórax se observó costilla cervical bilateral, llevándose a cabo resección de costilla cervical derecha, sin mejoría de la pérdida de fuerza probablemente por clínica de larga data.

**Conclusiones**: El síndrome de desfiladero torácico superior tiene como causas frecuentes estructuras anómalas como costillas accesorias, bandas fibrosas o mega-apófisis transversas de C7. Es importante buscar estas estructuras que pueden pasar inadvertidas. Una rápida actuación en estos casos puede mejorar el pronóstico por lo que es importante su sospecha en pacientes jóvenes.

**PO-30.- Síndrome de Bruns-Garland, a Propósito de un Caso**

Lara Mauri Fábrega^1^, Teresa Fábregas Ruano^1^, Nerea Armario Redondo^1^, Ana Rodríguez Reguera^1^

^1^H. U. Jerez de la Frontera, 11407 Cádiz, España

**Introducción**: La radiculoplexopatía lumbosacra, síndrome de Bruns-Garland o amiotrofia diabética de miembro inferior, es una complicación poco frecuente del sistema nervioso periférico en pacientes con metabolopatías como la diabetes.

**Material y Método**: Mujer de 63 años con diabetes mellitus tipo 2 mal controlada de más de veinte años de evolución, que ingresa por cuadro de dolor intenso, paresia e hipoestesia proximal de miembro inferior derecho, de inicio agudo y de seis meses de evolución a su ingreso. A la exploración, presentaba atrofia y paresia de la musculatura proximal de pierna derecha 2–3/5, así como hipoestesia en cara anterior de muslo. Se solicitaron analítica, resonancia magnética de pelvis y columna lumbosacra con contraste, estudio neurofisiológico, panel de miopatías y biopsia glútea.

**Resultados**: Los estudios solicitados no mostraron resultados patológicos destacables con excepción del estudio neurofisiológico, que evidenció la presencia de una polineuropatía axonal sensitivo-motora, sobre la que se objetivaban datos de ridículo-plexopatía lumbosacra. Se estableció el diagnóstico de síndrome Bruns-Garland, se inició tratamiento rehabilitador y se ajustó el tratamiento antidiabético.

**Conclusiones**: El síndrome Bruns-Graland es una enfermedad de naturaleza probablemente inflamatoria, favorecida por metablolopatías predisponentes. Afecta normalmente de forma unilateral a miembros inferiores. El tratamiento con corticoides e inmunoglobulinas así como la fisioterapia pueden condicionar una mejoría parcial, siendo excepcional la recuperación completa.

**PO-31.- Experiencia con Perampanel en el Tratamiento de la Encefalopatía Post-Anóxica con Mioclonías: Síndrome de Lance-Adams. A Propósito de un Caso**

Kevin Jiménez Ureña^1^, Noelia Guerrero Carmona^1^, Julia Pinedo Córdoba^1^, Andrea Irene Cienfuegos Fernández^1^, María Fernández Recio^1^

^1^H. U. Virgen de Valme, 41014 Sevilla, España

Mujer de 18 años que presenta parada cardiorrespiratoria extrahospitalaria por shock anafiláctico, siendo atendida, recuperada, trasladada e ingresada en UCI de nuestro hospital. Como antecedentes personales destacaba ausencia de hábito tabáquico, asma persistente moderada con numerosas exacerbaciones, estudiada por alergología por sospecha de alergia a huevo y leche, en tratamiento con salbutamol a demanda y bilastina. Tras un día en situación de sedoanalgesia e intubación orotraqueal, se decide retirar dichas medidas mostrando entonces la paciente confusión y mioclonías generalizadas. Ante la sospecha de estatus epiléptico se inicia midazolam y levetiracetam, sin mejoría clínica. Se progresa añadiendo fenitoína y ácido valproico manteniendo pobre respuesta por lo que se realiza interconsulta con neurología. A nuestra valoración la paciente presenta mioclonías generalizadas constantes exacerbadas por el movimiento. Se realiza EEG en el que se aprecian ondas agudas generalizadas compatibles con actividad epileptiforme o con patrón electromiográfico por las mioclonías constantes con afectación facial. Se suspende fenitoína y se inicia clonazepam con mejoría inicial. La paciente pasa a planta regular manteniendo mioclonías incapacitantes y amnesia anterógrada que afectaba a todo el período de ingreso. Se inicia entonces perampanel a dosis de 4 mg/24 h con mejoría clínica notable. La paciente en los días posteriores mejora la capacidad de hablar (dificultada por mioclonías faciales), en miembros superiores las mioclonías aparecen tan sólo al final del movimiento y es capaz de mantener bipedestación con andador. Tras mejoría progresiva de la amnesia se decide alta a domicilio manteniendo terapia combinada con perampanel, clonazepam y levetiracetam.

**PO-32.- Afectación Atípica en Órgano Diana en Paciente con Hipotensión Arterial Farmacológica**

Jorge Román Rueda^1^, Carlota Villar Rodríguez^1^, Antonio Cristóbal Luque Ambrosiani^1^, Luis Fernández Espigares^1^, Mikel Salgado Irazábal^1^, Leire Ainz Gómez^1^

^1^H. U. Virgen del Rocío, 41013 Sevilla, España

**Objetivos**: Las lesiones isquémicas espinales tras cirugías suelen ser próximas a la intervención y secundarias a hipoperfusión. Reportamos un caso de infarto medular tardío, al transcurrir un mes tras tratamiento endovascular sobre la aorta.

**Material y Métodos**: Varón 62 años con hipertensión arterial (HTA) severa secundaria a coartación de aorta postductal, en politerapia con múltiples antihipertensivos. Se somete a implantación de endoprótesis en arco aórtico como tratamiento de la coartación en septiembre de 2022. Al alta, mantiene tratamiento con fármacos hipotensores habituales.

**Resultados**: En octubre de 2022, consulta por cuadro brusco de dolor dorsal intenso, debilidad y parestesias en los cuatro miembros, retención urinaria y dificultad para la defecación. Presenta cifras de tensión arterial bajas, en torno a 80/40 mmHg, habituales en domicilio durante las semanas previas. Se realiza RM urgente de columna sin hallazgos significativos, ingresando con sospecha de isquemia aguda medular. A las 72 horas se repite una nueva RM en la que se confirma la presencia de un infarto medular longitudinalmente extenso en el territorio de la arteria espinal anterior. Durante su estancia se descartan etiologías embolígenas alternativas, por lo que el evento se atribuye a la hipoperfusión medular secundaria a la intervención, junto a la hipotensión mantenida.

**Conclusiones**: Los infartos medulares tras cirugías se reportan en torno a 4,5% hasta 30 días tras intervención. La repercusión sobre la circulación colateral en este tipo de intervenciones obliga a un especial manejo, que priorice una adecuada perfusión medular, así como seguimiento de la TA para evitar complicaciones.

**PO-33.- Evolución Radiológica de Lesiones Secundarias a Estatus Epiléptico: A Propósito de un Caso**

Carmen Ortega Hiraldo^1^, Trinidad Sanjuán Pérez^1^, Alba Aguilar Monge^1^, Ana Gómez González^1^, Marta Vicente Domínguez^1^

^1^H. U. Virgen de la Victoria, 29010 Málaga, España

**Introducción**: El estatus epiléptico (EE) es una situación urgente resultante de un defecto en los mecanismos de terminación de las crisis comiciales. Su etiología es variada y la RMN es una herramienta fundamental para determinarla, mostrando también cambios producidos por la propia actividad epiléptica.

**Material y Métodos**: Caso clínico de un paciente en situación de estatus no convulsivo (EENC) provocado por meningioma y lesiones secundarias al estatus en RMN, con resolución de estas en control evolutivo de neuroimagen.

**Resultados**: Varón, 79 años, sin antecedentes de epilepsia, atendido de forma urgente con situación clínica compatible con EENC. Se administran LEV y LCM con resolución sintomática y en TC urgente se objetiva lesión frontotemporal derecha compatible con meningioma de gran tamaño. Durante hospitalización, se realiza RMN que muestra lesiones hiperintensas en FLAIR que restringen en difusión, cerebelosas simétricas, y en corteza frontotemporal izquierda. Se realiza estudio de extensión, punción lumbar y análisis sin alteraciones. En EEG descargas agudas en región frontal izquierda, y ondas trifásicas frontotemporales derechas. En RMN de control, a los 14 días, y en control postquirúrgico, al mes, se mantiene una leve hiperintensidad FLAIR cerebelosa bilateral sin traducción en las secuencias de difusión, con desaparición de la hiperintensidad cortical previa.

**Conclusiones**: Los mecanismos subyacentes al EE suponen cambios en la perfusión cerebral que se traducen en alteraciones en la neuroimagen. Las secuencias de difusión son las más sensibles para detectar las anormalidades periictales, que pueden encontrarse en la regiones corticosubcorticales en relación con el área epileptógena y en localizaciones remotas por posibles mecanismos de diasquisis.

**PO-34.- Ictus Hemorrágico Bitalámico Recurrente y Sincrónico a Defecto Severo en la Hemostasia Primaria, Alfa Talasemia Minor e Hipertensión Arterial Mal Controlada: Reporte de un Caso**

Luis Manzano Hernández^1^, Mikel Salgado Irazábal^1^, Antonio Luque Ambrosiani^1^, Pablo Gómez López^1^, Manuel Medina Rodríguez^1^

^1^H. U. Virgen del Rocío, 41013 Sevilla, España

Presentamos el caso de un hombre nigeriano de 56 años con AP de Ictus hemorrágico talámico derecho de causa hipertensiva hace 3 meses. Clínica residual motora y sensitiva en miembros izquierdos. De forma súbita presenta debilidad en el hemicuerpo derecho, afasia y tensiones elevadas. TC de cráneo con colección hemática en tálamo izquierdo de densidad heterogénea con apertura a ventrículo lateral. Hematoma talámico derecho crónico con foco hemorrágico frontal. Se excluye patologías vasculares intracraneales. El estudio de cribado de la función plaquetaria (PFA-100) fue un aliado al identificar una alteración severa de la hemostasia primaria sin déficit cuantitativo del Factor Von Willebrand. Fue necesaria la transfusión de concentrados plaquetarios. La clínica debut de este defecto fue exclusivamente neurológica. En el ingreso se diagnosticó de Alfa-talasemia minor, SAOS e Hipertrófica ventricular izquierda concéntrica. La Hipertensión Arterial explica la primera causa de Ictus hemorrágico en territorio cerebral profundo y su morbi-mortalidad depende directamente del crecimiento activo del hematoma. Ante la nueva clínica neurológica debe sospecharse una etiología similar a la anterior y mala adherencia al tratamiento. En la población negra se indicará de inicio doble Anti-HTA con Calcioantagonista dihidropiridínico + Antagonista de los receptores de angiotensina II o Diurético tiazídico. En los Ictus hemorrágicos recurrentes se pensará en el debut de trastornos de la hemostasia primaria sin plaquetopenia. La enfermedad del FvW es la alteración congénita más frecuente de la coagulación. En el tipo II (a y b) la alteración estará en su estructura molecular como es el caso de nuestro paciente.

**PO-35.- Un Caso de Síndrome Medular Agudo por Dorsiflexión del Tronco Viendo la TV**

Julia Pinedo Córdoba^1^, María del Carmen García López^1^, Noelia Guerrero^1^, Kevin Jiménez Ureña^1^, Andrea Cienfuegos^1^

^1^H. U. Virgen de Valme, 41014 Sevilla, España

Describimos un caso que debutó inicialmente como debilidad en MMII recurrente en relación a un mecanismo postural hasta provocar de forma permanente un síndrome medular agudo por mielopatía dorsal longitudinalmente extensa secundaria a fístula A-V. Debutó como un síndrome de arteria espinal anterior, pero en cuanto a la etiología la fístula arterio-venosa espinal dural es una entidad patológica muy infrecuente. Varón de 57 años desde hace 5 meses presenta episodios autolimitados de debilidad en MMII tras mantener posturas de dorsiflexión del tronco con resolución completa al volver a reposo y la extensión del tronco. Desde entonces estos episodios han adquirido mayor entidad. Acude a Urgencias por presentar tras estar horas sentado en el sillón en posición de “V” cuadro de debilidad en MMII y práctica anestesia desde región infraumbilical. Exploración: Paraplejia de MMII. REM abolidos de forma global en MMII. Hipoestesia termoalgésica en MMII con nivel sensitivo altura de D10. Sensibilidad vibratoria y posicional conservada. RM columna: hiperintensidad centro medular desde D4 a D12-L1 dilatación y tortuosidad de las venas peri medulares que sugiere fistula arteriovenosa/malformación vascular espinal tipo I. Arteriografía confirma la existencia de fístula A-V. Evolución: recibió corticoterapia por posibilidad inicial de patología inflamatoria. Tras la confirmación la fístula se intervino neuroquirúrgicamente. Actualmente mantiene bipedestación pero precisa silla de ruedas, está realizando tto rehabilitador. Reconocer esta patología puede prevenir un daño neurológico irreversible. En el caso de nuestro paciente la semiología medular aguda junto con el mecanismo postural y la neuroimagen permitieron el diagnóstico de esta entidad poco conocida.

**PO-36.- Migraña con Aura, Crisis Comiciales y Aneurisma Intracraneal en Paciente con Telangiectasia Hemorrágica Hereditaria**

María Begoña Gómez González^1^, Miriam Sillero Sánchez^1^, Nuria Rodríguez Fernández^1^, Beatriz Rosado Peña^1^, Lucía Moreno Vico^1^

^1^H. U. Puerto Real, 11510 Cádiz, España

**Objetivos**: Describir la evolución clínica y síntomas neurológicos sobrevenidos en una paciente con diagnóstico de Telangiectasia Hemorrágica Hereditaria.

**Material y Métodos**: Mujer de 28 años diagnosticada de Migraña con aura en 2011 y con normalidad de exploración y RM cerebral. En 2019, por antecedentes familiares, antecedentes personales de sangrado nasal y gingival y disnea con el esfuerzo, es estudiada por Neumología, diagnosticada de enfermedad de Rendu-Osler (confirmada genéticamente y con criterios clínicos) y sometida a embolización de malformaciones arteriovenosas pulmonares, con mejoría sintomática.

**Resultados**: En 2020 sufre un posible síncope convulsivo, que se siguió de crisis comiciales de inicio focal en los miembros derechos. Una angioTAC mostró un aneurisma en carótida interna izquierda intracraneal, valorado por Neurocirugía (actitud expectante) y en el Electroencefalograma había actividad epileptiforme hemisférica izquierda. Fue tratada con levetiracetam, sustituido por brivaracetam por persistencia de crisis.

**Conclusiones**: La Telangiectasia Hemorrágica Hereditaria representa una enfermedad sistémica que puede afectar a la vasculatura de diferentes órganos. En el sistema nervioso se ha relacionado fundamentalmente con isquemia/hemorragia, y algo menos con crisis comiciales. En nuestra paciente convergen diferentes patologías neurológicas cuya relación con esa enfermedad, que no puede establecerse con certeza, debe al menos ser contemplada como posible.

**PO-37.- Diagnóstico Concomitante de Síndrome de Miller Fisher y Síndrome de Sjögren: A Propósito de un Caso**

Cuenca Relinque Adelaida^1^, Arzalluz Luque Joaquín^1^, Pérez Vizuete Isidro^1^, Torres Moral Alejandro^1^, Ortega Ortega Francisco Fernando^1^, Calle Serrano Marta^1^, Ben Yelun Insenser Miriam^1^, Barragán Prieto Ana^1^, Navarro Mascarell Guillermo^1^

^1^H. U. Virgen Macarena, 41009 Sevilla, España

**Objetivos**: Se presenta un caso de Síndrome de Miller-Fisher (SMF) a raíz del cual, de forma incidental durante el estudio etiológico, se diagnostica Síndrome de Sjögren (SS).

**Material y Métodos**: Varón de 25 años, sin antecedentes de interés, que presenta de forma subaguda diplopía horizontal (ojos congelados), seguida de inestabilidad de la marcha. Niega síntomas infecciosos días previos u otros síntomas neurológicos. A la exploración presenta oftalmoplejía, arreflexia generalizada con fuerza y sensibilidad conservadas, dismetría dedo-nariz y talón-rodilla bilateral, marcha inestable con aumento de base de sustentación y tándem imposible.

**Resultado**: Como estudio complementario, se realizó neuroimagen completa y estudio de LCR con citobioquímica, autoinmunidad y microbiología normales, ENG con ausencia de respuesta H y analítica con anticuerpos anti-gangliósido-GQ1b, con serologías y autoinmunidad negativas, excepto positividad de anti-SSA/Ro. A raíz de este hallazgo se realizaron test de Schimer y biopsia de glándulas salivares menores patológicos diagnosticándose SS. Se trató con inmunoglobulinas y plasmaféresis con evolución favorable quedando de forma residual oftalmoplejía. Se propuso tratamiento con corticoides el cual el paciente rechazó.

**Conclusiones**: El paciente presenta la triada y los anticuerpos clásicos anti-GQ1b del SMF, enfermedad infrecuente cuya fisiopatología es el mimetismo molecular; los anticuerpos se producen contra los componentes de los patógenos similares a los gangliósidos que son consecuentemente atacados. El SS es una enfermedad autoinmunitaria sistémica con disfunción de las glándulas exocrinas. Pese a fisiopatología diferenciada comparten etiología inmunomediada y se investiga su correlación y el beneficio de terapia corticoidea no descrito hasta ahora para el SMF.

**PO-38.- Contra Todo Pronóstico: La Descripción de un Caso de Disección Carotídea Que se Presenta Como Síndrome de Horner Sin Evento Cerebrovascular**

Cienfuegos Fernández Andrea Irene^1^, Córdova Infantes María del Rocío^1^, García López María del Carmen^1^, Guerrero Carmona Noelia^1^, Jiménez Ureña Kevin^1^, Pinedo Córdoba Julia^1^

^1^H. U. Virgen de Valme, 41014 Sevilla, España

**Objetivos**: La disección carotídea es causa frecuente de ictus isquémico en pacientes menores de 50 años. La triada de presentación típica en orden de frecuencia es cefalea/cervicalgia (65–95%), AIT/ictus (>50%) y el síndrome de Horner incompleto (25%). Describimos el caso de un paciente con una forma de presentación clínica poco habitual.

**Material y Métodos**: Varón de 43 años ex-fumador y sin otros factores de riesgo cardiovascular, que acude por miosis. En la anamnesis dirigida relataba dolor cervical derecho hacía una semana mientras montaba en bicicleta y alteraciones visuales. A la exploración neurológica ptosis palpebral y miosis en ojo derecho (OD) sin otros déficits asociados y cifras tensionales altas. Se solicitó analítica, radiografía de tórax, ECG, TAC craneal, angio-TAC craneal y de TSA y RMN craneal.

**Resultados**: Analítica, radiografía y TAC fueron normales. En angio-TC/TSA presentaba disección de la arteria carótida interna derecha desde el segmento cervical hasta el intrapetroso con la luz permeable. En angio-TC craneal no hubo oclusión de ACM, ACA o ACP. Durante su ingreso en planta se realizó una RMN craneal, que también fue normal. No presentó nueva focalidad durante el ingreso y precisó tratamiento retiniano en OD.

**Conclusión**: Pese a que existe una alta frecuencia de aparición de AIT/ictus en la disección carotídea y que el riesgo de presentarlo en las semanas siguientes es de hasta 13%, existen casos, como el nuestro, que son paucisintomáticos con disecciones extensas. La HTA no diagnosticada ni tratada a tiempo parece haber sido un factor importante en la fisiopatología de esta entidad.

**PO-39.- Neuro-Behçet, Lesiones Multifocales en la Unión Mesodiencefálica que Preceden a Manifestaciones Inflamatorias Sistémicas: Reporte de un Caso**

Luis Manzano Hernández^1^, Antonio Cristóbal Luque-Ambrosiani^1^, Francisco Hernández-Chamorro^1^, Francisco Javier Gómez-Fernández^1^, Alfredo Palomino García^1^, Francisco José Hernández Ramos^1^

^1^H. U. Virgen del Rocío, 41013 Sevilla, España

Presentamos el caso de una mujer de 69 años sin AP. Acude a Urgencias por bradipsiquia y bradilalia. Parafasias. Pupila de Adie izquierda. Hipoestesia facial izquierda y hemiparesia izquierda. Dismetría izquierda. Dolor abdominal difuso con estreñimiento/diarrea intermitente de 2 meses. Reflejos miotáticos izquierdos exaltados, reflejo cutáneo-plantar-izquierdo-extensor. Romberg positivo. Marcha con aumento en base. No irritación meníngea. Fenómeno Patergia negativo. Ingresó en Neurología afebril, niega viajes, infecciones recientes y úlceras genitales/orales. Tres LCR con ligera proteinorraquia/pleocitosis-linfocitaria leve. Bandas, citometría, autoanticuerpos y HLA-B51 negativos. RM con lesiones extensas hipointensas en T1, hiperintensa en FLAIR a nivel del troncoencéfalo, diencéfalo y cápsulo-ganglionar derecho con comportamiento heterogéneo tras contraste, propia de Neuro-Behçet. Descartada patología reumatológica/infecciosa/inflamatoria/oncológica, es diagnosticada como Probable Síndrome de Neuro-Behcet por criterios de la ICR, parciales de EULAR. Administramos 1 gr de Metilprednisolona IV (5dias) Sin patología abdominal es dada de alta con Prednisona 60 mg/día y Micofenolato 250 mg/12 h (posterior 500 mg/12 h) con magnifica respuesta. PET-TC ambulatoria con lesiones típicas del Síndrome-Behcet sistémico: paniculitis mesentérica con perforación-fístula intestinal. El SB es una enfermedad multisistémica inflamatoria idiopática, crónica y recurrente. El SN-B está entre el 3–9% de los SB. Aparece después de la tercera-cuarta década. La clínica neurológica transcurre un período de 5–6 años después de los sistémicos, aunque pueden aparecer al mismo tiempo (7%) o precederlos (3%), siendo esta última población un verdadero desafío para el neurólogo clínico, como el caso que presentamos. La clínica suele justificarse por lesiones del RM. Es una entidad con poca homogeneidad diagnostica-terapéutica en auge académico, en nuestro caso con magníficos resultados.

**PO-40.- Cuando los Hallazgos Clínicos Asociados Dan la Clave Para el Estudio Genético: A Propósito de un Caso de Neuropatía Hereditaria**

Luque Ambrosiani Antonio Cristóbal^1^, Salgado Irazábal Mikel^1^, Hernández Chamorro Francisco José^1^, Villagrán Sancho Diego^1^, Gómez Fernández Francisco Javier^1^

^1^H. U. Virgen del Rocío, 41013 Sevilla, España

**Objetivos**: La causa más frecuente de neuropatía hereditaria es la enfermedad de Charcot-Marie-Tooth (CMT), presentando un amplio espectro clínico, por lo que es relevante estudiar los hallazgos clínicos asociados para poder dirigir el estudio genético e interpretar correctamente sus resultados. Presentamos un caso de un varón con lipomatosis y neuropatía axonal en el que su combinación permitió el diagnóstico.

**Material y Métodos**: Descripción de un caso clínico y revisión bibliográfica de los estudios recientes sobre lipomatosis y neuropatías.

**Resultados**: Varón de 65 años, con antecedentes familiares de padres consanguíneos, así como un hermano con lipomatosis y “problemas óseos”; como antecedente personal lipomatosis múltiple. Consulta por dolor y calambres en ambos pies, de años de evolución, así como por presentar pies cavos. Cuando es valorado en Neurología, se aprecian lipomas múltiples de predominio en tronco superior, así como paresia distal de MMII, con mayor afectación de la musculatura peroneal, con hipopalestesia distal e hiporreflexia rotuliana y arreflexia aquílea, con marcha taloneante y atrofia distal de MMII. Se realiza ENMG con hallazgo de neuropatía sensitivo-motora axonal, con autoinmunidad, serologías y analítica general normales. Por el hallazgo de lipomatosis múltiple y neuropatía axonal, se solicita panel de CMT, incluyendo MFN2, el cual resulta positivo, con cambio de secuencia c.2119C>T (p.Trp740Ser, rs28940292) en homocigosis en dicho gen.

**Conclusiones**: El estudio de las neuropatías hereditarias requiere de la búsqueda de signos clínicos asociados para acotar el diagnóstico, pues al dirigir el estudio genético correctamente, la interpretación de sus resultados se convierte en una tarea más sencilla, evitando la sobre interpretación de estos.

**PO-41.- De la Encefalitis a la Migraña: Una Anamnesis Decisiva**

Pablo Hernández Vitorique^1^, Mariam Nadia Afkir Ortega^1^, Virginia Delgado Gil^1^, Minerva Mañez Sierra^1^

^1^H. U. Virgen de la Victoria, 29010 Málaga, España

**Introducción**: La migraña hemipléjica familiar (MHF) es una variante de la migraña con aura que, en ocasiones, se puede presentar como un trastorno del lenguaje severo que puede confundirse con procesos vasculares o encefalíticos, donde solo los antecedentes familiares marcan la diferencia.

**Material y Métodos**: Varón de 28 años que cursa con episodio transitorio de afasia severa diagnosticado finalmente de MHF.

**Resultados**: Paciente traído a urgencias por episodio brusco de alteración global del lenguaje. A la exploración mutista y sin comprender órdenes, resto de exploración neurológica sin focalidad. Se activa código ictus con Tc, AngioTc y perfusión normales. Ante la persistencia de los síntomas se realiza punción lumbar y se comienza tratamiento empírico con aciclovir y antibióticos para cubrir principales patógenos de encefalitis aguda. La bioquímica, cultivos y PCR de virus en líquido cefalorraquídeo no mostraron hallazgos patológicos. El paciente estaba a la espera de ser ingresado cuando aparecen familiares y su madre comenta en anamnesis dirigida que tanto ella como la tía del paciente sufren episodios de cefalea con una alteración del lenguaje similar y están diagnosticadas de MHF. Tras 24h de observación el paciente se recupera completamente permaneciendo asintomático. Se realizó RMN de cráneo normal y se diagnostica eventualmente de MHF.

**Conclusiones**: Aunque la MHF es una patología estadísticamente infrecuente, debemos tenerla en cuenta ante aparición de focalidad neurológica abrupta pues, en urgencias, solo podremos diagnosticarla con una buena anamnesis dirigida al paciente o, en su defecto, a los familiares.

**PO-42.- A Propósito de un Caso de Aman**

Ortega Ortega Fernando Francisco^1^, Cuenca Relinque Adelaida^1^, Calle Serrano Marta^1^, Conde Velasco Coral^1^, Montaner Villalonga Joan^1^

^1^H. U. Virgen Macarena, 41009 Sevilla, España

**Objetivos**: Ilustrar sobre la presentación clínica de la variante axonal motora (AMAN) del Síndrome Guillain Barré, poco frecuente en nuestro medio con una progresión más y peor pronóstico funcional que la variante clásica.

**Material y Métodos**: Descripción de un caso de Síndrome de Guillain Barré variante axonal.

**Resultados**: Varón de 54 años con DM tipo 2, consulta por debilidad de cuatro extremidades de menos de 24 horas de evolución. Al dificultarle continuar en su trabajo, decide irse a su casa. Empeora a lo largo del día, con dificultad en la deambulación y al manipular objetos. La semana previa presentó diarrea acompañado de moco, y distermia. Niega clínica troncular, esfinteriana o respiratoria. No viajes al extranjero ni consumo de alimentos en mal estado recientes. A la exploración, presenta debilidad motora severa de predominio distal en miembros superiores y proximal en inferiores, con arreflexia en miembros superiores e hiporreflexia en inferiores. No alteraciones de la sensibilidad. Hipopalestesia leve en miembros inferiores hasta espina tibial. Ante la sospecha de Poliradiculoneuropatía aguda, ingresa en Neurología para estudio. Entre las pruebas realizadas, a destacar LCR sin disociación albuminocitológica, anticuerpos anti GM1, antiGD1a y antiGD1b positivos y estudio neurofisiológico con datos de polirradiculoneuropatía axonal y predominio motor. Como medidas terapéuticas, recibe cuidados de fisioterapia y 8 sesiones de plasmaféresis en dos ciclos, junto a 5 infusiones de Inmunoglobulinas.

**Conclusiones**: La variante axonal motora del SGB es poco frecuente en nuestro medio, suele precederse por cuadros gastrointestinales con aislamiento de Campylobacter jejunii y clínicamente predomina la afectación motora con escasa clínica sensitiva y sin afectación de pares craneales. La progresión clínica hasta alcanzar el NADIR suele ser más rápida que en la AIDP, y el pronóstico empeora en aquellos casos con degeneración axonal extensa.

**PO-43.- Toxina Botulínica: Tratamiento en Pacientes con Neuralgia Occipital Refractaria**

Cuenca Relinque Adelaida^1^, Montero Ramírez Emilio^1^, Calle Serrano Marta^1^, Ortega Ortega Fernando Francisco^1^, Viguera Romero Francisco Javier^1^

^1^H. U. Virgen Macarena, 41009 Sevilla, España

**Objetivos**: Presentar el caso de una paciente con neuralgia occipital refractaria con respuesta favorable a la infiltración con toxina botulínica.

**Material y Método**: Mujer de 65 años, hipertensa y dislipémica, que presenta dolor punzante con sensación de disestesias en la parte occipital izquierda, en la exploración presenta dolor a la palpación del nervio occipital mayor.

**Resultado**: Se diagnóstico, al cumplir los criterios, de neuralgia occipital y mediante pruebas complementarias se descartó causas subyacentes. Se trató en un inicio con medidas conservadoras y tratamiento farmacológico con duloxetina, eslicarbacepina y pregabalina, precisando infiltración de mepivacaína. Tras varias infiltraciones anestésicas con resultados subóptimos se decide buscar terapias alternativas como infiltración con toxina botulínica A, infiltrándose 40 U a nivel occipital y 10 U suboccipital, que tuvo como resultado un descenso en la intensidad y la frecuencia de la neuralgia a los tres meses de la infiltración, sin efectos secundarios.

**Conclusiones**: La neuralgia occipital es tras la neuralgia trigeminal la más frecuente, con un gran impacto en la calidad de vida de los pacientes. La mayoría responden de forma adecuada al tratamiento farmacológico y a la infiltración anestésica pero a menudo se presentan casos farmacorresistentes que precisan de terapias alternativas, como puede ser la toxina botulínica, la cual en diversos estudios como tratamiento para la neuralgia refractaria está demostrando resultados prometedores disminuyendo la intensidad y la frecuencia de la neuralgia y aumentando la calidad de vida , pero aún quedan muchas cuestiones por responder que terminen de situar su papel en dicha enfermedad.

**PO-44.- Síndrome de Nieve Visual. A Propósito de un Caso**

García Ortiz Manuel^1^, Molinero Marcos Alejandro^1^, Acosta de los Reyes María^1^, Coronado Puerto Cecilia^1^, Serrano García Irene^1^, Ana Gómez Roldós^1^

^1^H. U. Puerta del Mar, 11009 Cádiz, España

**Objetivos**: El SNV es un trastorno del Sistema Nervioso Central para cuya fisiopatología se sugiere que existe una hiperexcitabilidad del córtex visual y una disfunción del procesamiento visual de orden superior, sin esclarecer la localización topográfica de la lesión (córtex visual primario o córtex visual asociativo). Se caracteriza por la visión de pequeños puntos blancos y negros centelleantes en la totalidad del campo visual durante más de 3 meses, asociado al menos a 2 de los siguientes síntomas: Palinopsia. Nictalopía. Fotofobia. Otros síntomas visuales positivos tales como miodesopsias, metamorfopsias o halos de luz en torno a estímulos luminosos. Nuestro objetivo es describir un caso de migraña con síntomas vertiginosos asociados a SNV, en el que existe una excelente respuesta clínica a la flunarizina por parte de ambas entidades.

**Material y Métodos**: Varón de 41 años sin antecedentes de interés, ni consumo de tóxicos que presenta cefalea hemicraneal izquierda de carácter opresivo-pulsátil junto a mareos e inestabilidad de la marcha de 5 años de evolución. Asocia episodios de alteraciones visuales de 2–3 horas de duración que define como puntos centelleantes en todo el campo visual y visión borrosa en espacio poco iluminados sin relación con su cefalea. Además, describe fotofobia concomitante al desarrollo de estos síntomas visuales. Exploración neurológica normal. Pruebas complementarias realizadas incluyendo EEG y RM cerebral sin alteraciones. La valoración por Oftalmología y Otorrinolaringología fue normal.

**Resultados**: El paciente presenta cefalea de características migrañosas junto a síntomas vestibulares asociados y de manera concomitante clínica compatible con SNV que no relaciona con su migraña. La relación entre el SNV y la migraña es controvertida. La migraña se considera una comorbilidad frecuente del SNV. No se han demostrado tratamientos efectivos, la lamotrigina ha sido el fármaco más empleado en las series de casos publicados. Nuestro caso ha presentado una respuesta óptima al tratamiento con flunarizina, tanto de los síntomas correspondientes al SNV como los síntomas migrañosos. Esta opción terapéutica no está descrita en este tipo de casos.

**Conclusión**: Se considera que el SNV es una entidad infradiagnosticada. Si bien su condición es de benignidad, puede ser especialmente limitante para la vida diaria del paciente debido a los síntomas visuales constantes que asocia. La flunarizina parece una opción razonable como tratamiento de migraña con síntomas vertiginosos y síndrome de nieve visual asociado.

**PO-45.- Disección Carotídea Intracraneal Tras Ejercicio de Crossfit: A Propósito de un Caso de Ictus Isquémico en Paciente Joven**

Ignacio Ruiz Salcedo^1^,Víctor Carmona Bravo^1^, Coral Conde Velasco^1^, Alexandra Rincón Valencia^1^

^1^H. U. Virgen Macarena, 41009 Sevilla, España

**Objetivos**: La disección carotídea está ampliamente descrita como causa de ictus isquémico en paciente joven. El principal mecanismo es de etiología traumática, habiéndose descrito factores no traumáticos como enfermedades de tejido conectivo. Se presenta el caso de una paciente joven con ictus isquémico por disección carotídea intracraneal (ACI) posiblemente secundario a ejercicio de Crossfit.

**Material y Métodos**: Mujer de 27 años con antecedentes de migraña sin aura que presenta una intensa cefalea hemicraneal derecha con sonofotofobia, ausencia de respuesta a analgesia, acompañada de déficit sensitivo-motor faciobraquial izquierdo, orientada en urgencias como crisis migrañosa con aura, con TC cráneo normal. Meses después, experimenta episodios de déficit sensitivo-motor queirocrural izquierdo autolimitados, sin cefalea. Se realiza RM cráneo y angioRM con evidencia de infarto isquémico crónico en territorio de arteria cerebral media derecha y suboclusión del segmento supraclinoideo de ACI ipsilateral.

**Resultados**: Tras descartar etiología cardioembólica y la mayoría de inhabituales, se realiza una arteriografía que muestra doble luz en ACI derecha terminal, compatible con disección. Por anamnesis dirigida, se identifica como causa más probable de la disección la práctica de Crossfit con movimientos de flexo-extensión cervical.

**Conclusiones**: Clásicamente, la disección carotídea traumática se ha vinculado con impacto cervical directo. Sin embargo, en las últimas décadas, se han descrito casos vinculados a la realización de diferentes deportes, incluso de bajo impacto y en ausencia de otros factores predisponentes. Destaca el Crossfit, que se ha identificado como potencial mecanismo traumático. Concienciar a la comunidad médica sobre esta asociación emergente podría ayudar a la detección precoz de esta patología neurovascular.

**PO-46.- El Sistema de Comunicación Aumentativa y Alternativa Como Individualización del Tratamiento en la Ela**

Ana Rodríguez Reguera^1^, Nerea Armario Redondo^1^, Estefanía Guisado Fernández^1^, Lara Mauri Fábrega^1^, Raúl Espinosa Rosso^1^

^1^H. U. Jerez de la Frontera, 11407 Cádiz, España

**Objetivos**: Mejorar la comunicación y reducir el aislamiento social.

**Material y Métodos**: Se realiza una consulta personalizada con la paciente en la unidad de foniatría y disfagia para valorar la capacidad de uso de los distintos tipos de SCAA (sistema de comunicación aumentativa y alternativa) y establecer el posible beneficio de estos dispositivos.

**Resultados**: La paciente no fue capaz de usar un teclado alfabético con intención comunicativa, por lo que se pasó a valorar el teclado simple symbol talker A con teclado querty, que fue capaz de entender y manejar de forma correcta. Se constató que no precisa de pulsador y mantiene un mejor uso táctil que ocular. Se consideró que la paciente podía beneficiarse de este sistema, así como su forma portátil UKITU.

**Conclusiones**: El SCAA permite una personalización del tratamiento rehabilitador del habla en los pacientes con ELA, permitiendo una mejor integración del paciente en su entorno y una mejor calidad de comunicación. Así mismo, puede revisarse su uso en sucesivas consultas para marcar el paso de la comunicación táctil a ocular dada la evolución natural de la enfermedad.

**PO-47.- ¿Encefalitis Transitoria o Estatus Epiléptico? Confusión Por Neuroimagen**

Pablo Hernández Vitorique^1^, Mariam Nadia Afkir Ortega^1^, Jorge Romero Godoy^1^, Minerva Mañez Sierra^1^

^1^H. U. Virgen de la Victoria, 29010 Málaga, España

**Introducción**: El estatus epiléptico es una emergencia neurológica que requiere diagnóstico y tratamiento precoz. Las características en neuroimagen del estatus epiléptico son diversas y, en ocasiones, pueden confundirse con otras patologías, como las encefalitis.

**Material y Métodos**: Varón de 79 años con neuroimagen patológica que confunde daños post-estatus con proceso encefalítico.

**Resultados**: Paciente presenta en urgencias disminución del nivel de conciencia y baja respuesta a estímulos. La tomografía computarizada (TC) de cráneo reveló una imagen hiperdensa con calcificaciones en el lóbulo temporal derecho, compatible con meningioma y electroencefalograma compatible con estatus epiléptico. Tras iniciar tratamiento con levetiracetam el paciente mejoró, permaneciendo asintomático. Se cursó ingreso y se realizó una RM cerebral que mostró, además de la lesión extraaxial temporal derecha compatible con meningioma atípico, lesiones con marcada restricción en la difusión en áreas cerebelosas simétricas, corticales temporales izquierdas y frontales derechas, con realces inespecíficos en cerebelo y troncoencefálico, sugiriendo un proceso encefalítico. Se completa estudio con análisis de sangre y estudio de líquido cefalorraquideo completo, además de BODY-TC sin hallazgos patológicos, el paciente continuó asintomático. No se encontraron hallazgos que justificaran el proceso encefalítico, por lo que se solicitó una nueva RM de control. En esta segunda resonancia, todas las lesiones observadas, excepto el meningioma, habían desaparecido, identificándose entonces como lesiones transitorias secundarias al propio estatus epiléptico.

**Conclusiones**: Durante el estatus epiléptico, el parénquima cerebral experimenta hipoxia y generación de material oxidativo, dañando zonas extremadamente sensibles como los ganglios basales y las células de Purkinje. Reconocer los cambios en neuroimagen post-estatus puede evitar confusiones diagnósticas y facilitar un manejo temprano y eficiente de estos pacientes.

**PO-48.- Amiotrofia Neurálgica en Miembros Inferiores: A Propósito de un Caso**

El Mouhajir Mohamed Hayar^1^, Rodrigo Herrero Silvia^1^, Torres Sánchez Gonzalo^1^

^1^H. U. Juan Ramón Jiménez, 21005 Huelva, España

**Introducción**: La plexopatía lumbar es una causa común de discapacidad en adultos. Siendo causada por factores estructurales como traumatismos, hematomas o neoplasias, o por factores no estructurales como plexopatías post-irradiación o vasculitis.

**Material y Métodos**: Varón de 76 años, hipertenso, dislipémico y obeso. No diabético. Consulta en urgencias por cuadro progresivo de 3 semanas que se inicia con intenso dolor en región anterolateral de muslo izquierdo requiriendo analgésicos intravenosos. Progresivamente inicia debilidad proximal en muslo izquierdo precisando ayuda mecánica para caminar. No afectación de miembros superiores ni esfínteres. Exploración: Amiotrofia cuádriceps izquierdo. Paresia de psoas 3/5 y cuádriceps 1/5 izquierdos. Resto de musculatura normal. Hipoalgesia parcheada en cara anterolateral (L2/L3) y territorio L4/L5 de MII. RCP flexora bilateral. No piramidalismo. Reconocimiento de dedos y artrocinética normal.

**Resultados**: Se descartó la causa estructural de la plexopatía mediante una RMN lumbar y un TC-body y se confirmó la radiculitis aguda del tronco superior del plexo lumbar izquierdo a través de ENG. ENG: Ausencia de respuestas de nervios peroneal superficial y safeno bilateral. EMG en reposo con actividad denervativa profusa en ILP, VL, VM y AL, de forma discreta en TA. Patrón contráctil muy deficitario en miotomos L2–L4. LCR: proteinorraquia 112.7 mg/dL, 25 células. Resto normal.

**Conclusiones**: En el caso de nuestro paciente, el curso agudo y progresivo de dolor seguido de debilidad y amiotrofia, junto con hipoestesia parcheada nos hizo pensar en la posibilidad de etiología idiopática de la plexopatía. Se debe considerar ante pacientes con dolor lumbar intenso de origen indeterminado, una vez descartadas causas estructurales.

**PO-49.- Pseudoaneurisma Cerebral Como Emancipador de Crisis Epilépticas por Esclerosis Mesial Idóneamente Controladas: A Propósito de un Caso**

Luis Manzano Hernández^1^, Kina Espinosa Jiménez Carlés^1^, Aurora González^1^, Elena Zapata Arrianza^1^

^1^H. U. Virgen del Rocío, 41013 Sevilla, España

Exponemos el caso de una mujer de 65 años con crisis epilépticas atribuidas a Esclerosis Mesial Temporal en RM con adecuado control farmacológico. Acude a urgencias por reincidencia de cuadros epilépticos ya descritos como episodios crisis tónico-clónicas generalizados y de desconexión del medio que persisten a pesar del control de desencadenantes. En neuroimágenes se observa una lesión ocupante de espacio intraparenquimatosa en región temporal medial izquierda, no visualizada en TC y RM anteriores. Se descartó neoplasia oculta sistémica. Arteriografía identifica estructura nodular de 8 mm × 4 mm sin arteria que pueda nutrirla, pero con relleno tardío central compatible con pseudoaneurisma parcialmente trombosado en territorio vascular poco común, correspondiente a Arteria Temporal Media rama de la ACP Izquierda. Al aislarla y extirparla quirúrgicamente se envía a AP. Se observa fragmentos pseudonodulares de superficie rugosa comprendidos entre 1,5 × 1 cm y 1 × 1 cm. Aspecto ampliamente hemorrágico, pseudoencapsulados confirmando el diagnostico. Los aneurismas son dilataciones focales y permanentes de una arteria. En el caso de los pseudoaneurismas, las capas del vaso son reemplazadas por tejido fibroso. Su diagnóstico de forma incidental ha aumentado debido a la mayor realización de pruebas de imagen y su buena calidad. La incidencia de aneurismas intracraneales es del 2–3% en ausencia de comorbilidades, aunque puede aumentar en mujeres, ancianos, fumadores o hipertensos. No obstante, los pseudoaneurismas tienen una proporción poblacional mucho más baja, y hasta el momento actual no hay reportes en ATM. Su etiología atraumática no está clara, aunque se cree que la hemodinámica juega un papel importante.

